# Integrated Single-Cell and Transcriptomic Analysis Suggests a Cuproptosis-Related Phenotype and Potential Therapeutic Target in Acute Pancreatitis

**DOI:** 10.3390/ijms27209004

**Published:** 2026-10-11

**Authors:** Qingfeng Xie, Maoqin He, Qi Wang, Weiqin Li

**Affiliations:** 1 The First School of Clinical Medicine, Southern Medical University, Guangzhou 510515, China; qingfengxie1022@163.com (Q.X.); hmqever@foxmail.com (M.H.); 2 Institute of Animal Nutrition and Feed Sciences, College of Animal Sciences, Zhejiang University, Hangzhou 310058, China

**Keywords:** acute pancreatitis, cuproptosis, single-cell transcriptomics, inflammatory microenvironment, tetrathiomolybdate

## Abstract

Acute pancreatitis (AP) is an acute inflammatory disorder of the exocrine pancreas with limited therapies. Whether cuproptosis, a copper-dependent cell death pathway, contributes to AP pathogenesis remains unknown. We integrated single-cell transcriptomics (GSE279876), single-cell transcriptomics (GSE188819), and human whole-blood transcriptomic data (GSE194331) to evaluate 13 cuproptosis-related genes across cell types, along with pseudotime trajectory, regulatory networks, and cell–cell communication. Findings were validated in caerulein-induced severe acute pancreatitis (SAP) mouse models and acinar cell injury using histopathology, electron microscopy, flow cytometry, immunofluorescence, and biochemical assays. Single-cell analysis showed selective activation of a cuproptosis-related signature in acinar cells, with all 13 genes upregulated, most notably *GIs* and *Fdx1*. The cuproptosis-related gene signature score increased along with pseudotime, and exploratory transcription factor (TF) and cell–cell communication analyses suggested enrichment of SPI1 and AP-1 (FOS/JUN), alongside MIF/CCL inflammatory signals, which was corroborated in independent datasets. In vitro and in vivo, pancreatic tissues and pancreatic acinar cells in SAP exhibited copper accumulation, FDX1/DLAT upregulation, mitochondrial shrinkage, and cristae loss, which are consistent with a cuproptosis-related phenotype, and were all attenuated by Tetrathiomolybdate (TTM). These findings suggest that the inflammatory microenvironment may be associated with selective activation of a cuproptosis-related program in acinar cells in AP, and that copper chelation with TTM attenuates pancreatic injury, indicating a potential therapeutic strategy targeting copper dyshomeostasis.

## 1. Introduction

Acute pancreatitis is an acute, critical illness of the digestive system with high incidence and mortality, and its core pathological event is the injury and death of pancreatic acinar cells [1,2,3]. Despite increasing insight into the pathophysiology of acute pancreatitis in recent years, clinically actionable targeted therapies remain unavailable, primarily because the diversity of acinar cell death modalities and the complexity of their regulatory networks have not been fully elucidated [4,5]. It has traditionally been believed that acinar cell necrosis is the dominant mode of cell death in acute pancreatitis. However, the subsequent identification of multiple programmed cell death modalities, including apoptosis, autophagy, pyroptosis, and ferroptosis, has prompted a multidimensional reappraisal of the pathological mechanisms of this disease [6,7]. Nevertheless, the role of cuproptosis, a recently defined mode of programmed cell death, in acute pancreatitis remains entirely unexplored.

Cuproptosis is a distinct cell death pathway driven by excessive copper accumulation and dependent on mitochondrial respiration. The core mechanism involves copper binding directly to lipoylated TCA cycle enzymes such as DLAT, leading to aberrant protein lipoylation and the loss of iron–sulfur cluster proteins, which ultimately causes mitochondrial dysfunction and cell death [8,9,10]. Since Tsvetkov et al. first described this pathway in *Science* (2022), cuproptosis has attracted immense interest across oncology, neurodegeneration, and inflammation research [11,12]. In inflammatory contexts, multiple connections between cuproptosis and cellular stress responses have been documented. Oxidative stress promotes the release of copper ions from metalloproteins, and the resulting increase in free copper further amplifies reactive oxygen species (ROS) generation [13,14]. Mitochondrial dysfunction is a central event in cuproptosis; copper accumulation impairs the activity of electron transport chain complexes, suppresses ATP synthesis, and disrupts the mitochondrial membrane potential [15,16]. Copper overload also activates endoplasmic reticulum (ER) stress, evidenced by upregulation of the unfolded protein response and disruption of intracellular calcium homeostasis [15]. Aberrant calcium release can activate calcineurin and inflammatory transcription pathways such as NF-κB [17]. Furthermore, aberrant modifications of cuproptosis-related proteins may serve as damage signals that promote NLRP3 inflammasome assembly and pro-inflammatory cytokine release [18,19]. These mechanisms have been preliminarily validated in other disease models, suggesting a complex bidirectional interplay between cuproptosis and inflammatory signaling.

It should be noted that cuproptosis is not only regulated by the intracellular copper metabolic network but may also be influenced by exogenous inflammatory signals, through which alterations in copper transporter expression, mitochondrial metabolic reprogramming, and elevated oxidative stress levels are induced; favorable cellular niches for cuproptosis are thereby created [20]. Notably, acute pancreatitis is characterized by intense local and systemic inflammatory responses; macrophage infiltration in pancreatic tissue, massive release of pro-inflammatory cytokines, and mitochondrial dysfunction are all highly overlapped with the triggering conditions of cuproptosis [21,22]. However, whether acinar cells may undergo a cuproptosis-related phenotype in association with the inflammatory microenvironment and whether susceptibility to cuproptosis differs among pancreatic cell types remain critical unanswered questions.

Therefore, in this study, we utilized single-cell transcriptome sequencing (scRNA-seq) and transcriptomic data from independent cohorts to systematically delineate the cell-type specificity, temporal dynamics, and transcriptional regulatory networks of cuproptosis in AP. Building on these analyses, we conducted multi-level experimental validation using animal and cellular models to provide new experimental evidence for the mechanistic understanding of acinar cell injury in AP from the perspective of cell death regulation.

## 2. Results

### 2.1. Single-Cell Transcriptomic Atlas of Acute Pancreatitis

**Figure 1:**
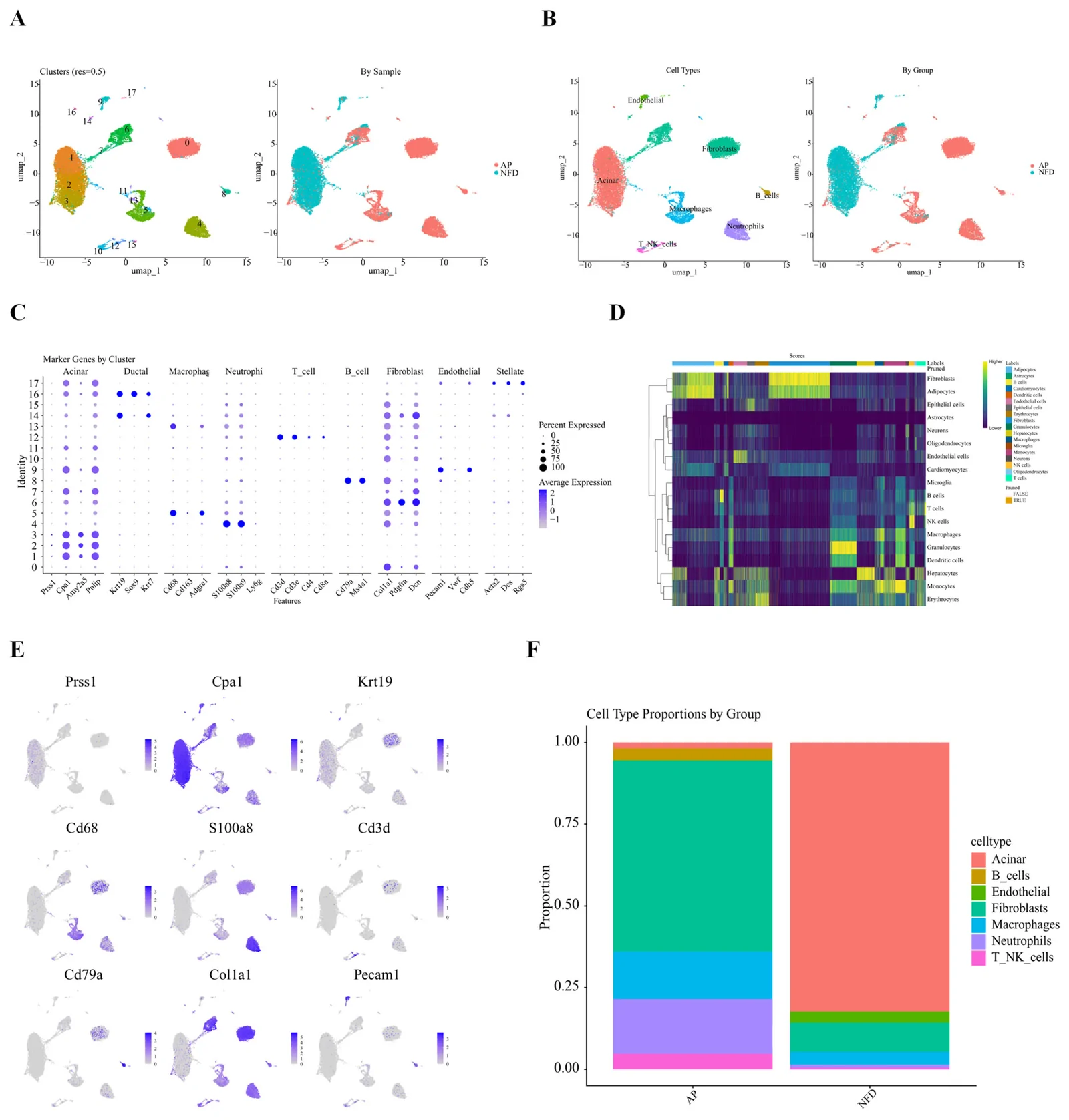
Single-cell transcriptomic atlas of acute pancreatitis. (**A**) UMAP dimensionality reduction clustering plot (resolution = 0.5), with the left panel colored by cell subpopulations and the right panel colored by sample origin, showing the distribution of four sample groups in the UMAP space. (**B**) UMAP plot of cell-type annotations, with the left panel indicating major cell types (acinar cells, ductal cells, neutrophils, macrophages, etc.) and the right panel colored by disease group, illustrating the distribution of each cell type across different groups. (**C**) Dot plot showing the expression levels of characteristic marker genes across cell subpopulations, where dot size represents the proportion of cells expressing the gene and color intensity indicates the average expression level. (**D**) Heatmap displaying the expression patterns of characteristic marker genes across cell subpopulations, with cell types annotated on the left and gene names on the right. (**E**) Feature plots showing the expression distribution of representative marker genes in the UMAP space, with color intensity indicating expression level. (**F**) Stacked bar plot of cell proportions, showing the percentage of each cell type across the four sample groups.

To systematically dissect how distinct cell types respond to cuproptosis in acute pancreatitis (AP), we analyzed the GSE279876 single-cell RNA-sequencing dataset. After quality control and Harmony-based batch correction, 36,794 high-quality cells were retained. The UMAP dimensionality reduction plot (resolution = 0.5) showed that cells from the normal-fat diet (NFD) and AP groups were evenly intermingled, indicating satisfactory batch correction (Figure 1A).

Cells were annotated into 10 major types—acinar cells, ductal cells, neutrophils, macrophages, fibroblasts, endothelial cells, T/NK cells, B cells, stellate cells, and others—by combining SingleR automatic annotation with manual curation based on canonical marker genes (Figure 1B). The dot plot displayed the expression of characteristic marker genes in each subpopulation, with high *Prss1*/*Cela1* in acinar cells, *Krt19* in ductal cells, *S100a8*/*S100a9* in neutrophils, and *Cd68/Apoe* in macrophages (Figure 1C). The heatmap further illustrated global expression patterns of these markers, consistent with the dot plot results (Figure 1D). Feature plots confirmed that *Prss1*, *Krt19*, and *S100a8* were specifically expressed in their respective subpopulations with minimal cross-expression (Figure 1E). A stacked bar plot of cell proportions revealed that the neutrophil fraction in the AP group increased substantially from approximately 2% in the NFD group to about 18%, accompanied by a rise in macrophages and a relative decline in acinar cells, consistent with the neutrophilic infiltration typical of acute pancreatitis (Figure 1F).

### 2.2. Cell-Type-Specific Activation of the Cuproptosis Pathway

**Figure 2:**
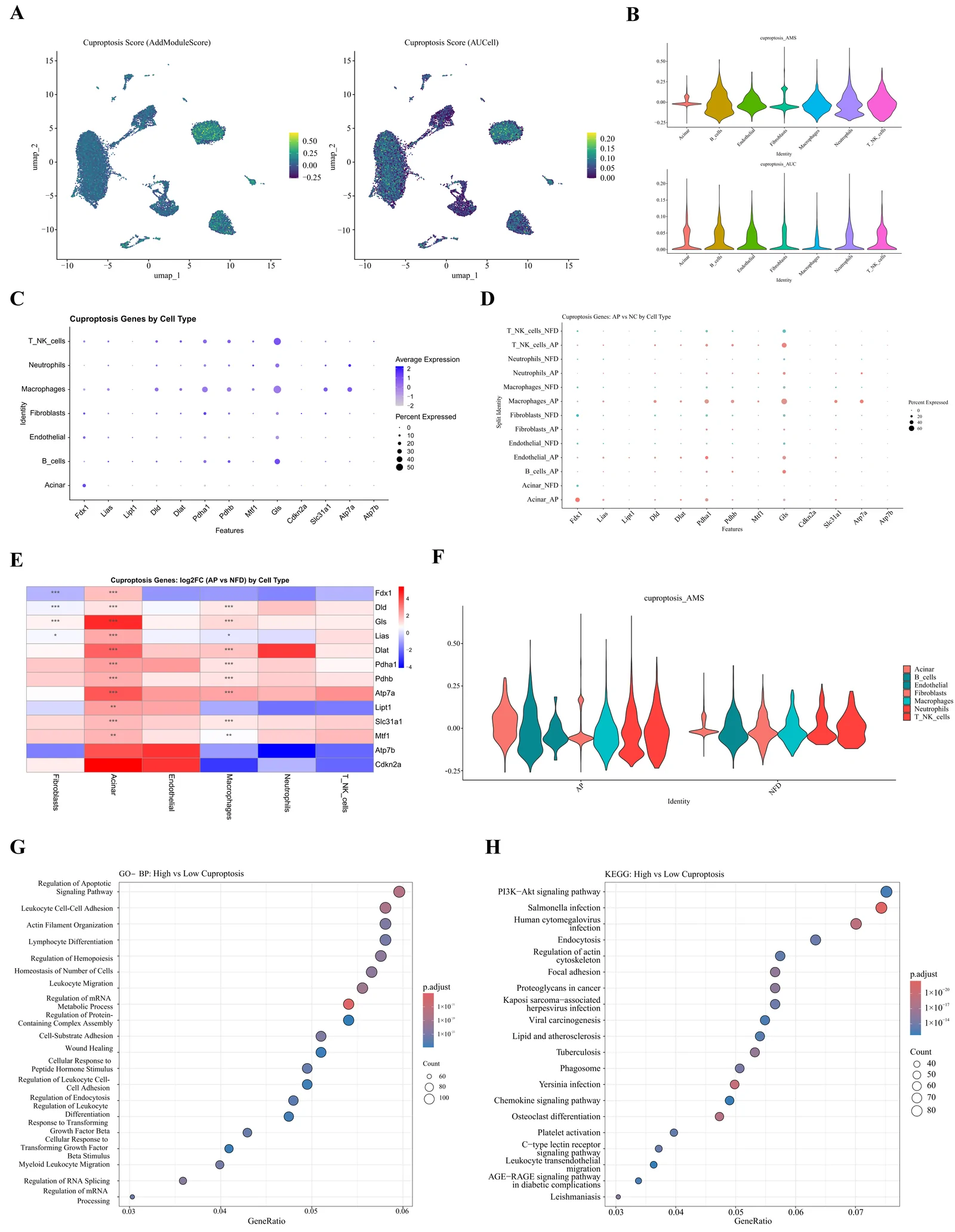
Cell-type-specific activation of the cuproptosis pathway. (**A**) UMAP dimensionality reduction overlay plot showing the cuproptosis pathway activity score for each cell, as evaluated by the AUCell algorithm, with color intensity reflecting pathway activity level. (**B**) Violin plots displaying the distribution of cuproptosis pathway scores across various cell types, with the *y*-axis representing pathway scores and the *x*-axis indicating different cell types. (**C**) Bubble plot illustrating the average expression levels of 13 core cuproptosis-related genes across cell types, where dot size represents the proportion of cells expressing the gene and color depth denotes the average expression level. (**D**) Split bubble plot showing the expression levels of cuproptosis genes specifically in acinar cells, stratified by experimental group.Dot size represents the proportion of cells expressing the gene, and color depth denotes the average expression level. (**E**) Heatmap depicting the expression patterns of the 13 core cuproptosis-related genes across cell types, with red indicating high expression and blue indicating low expression. Asterisks indicate statistical significance: * *p* < 0.05, ** *p* < 0.01, *** *p* < 0.001 (**F**) Grouped violin plots showing the differences in cuproptosis pathway score distribution among acinar cells across experimental groups. (**G**) Bubble plot of Gene Ontology (GO) biological process (BP) enrichment analysis, displaying the top 20 biological pathways enriched among differentially expressed genes between high- and low-cuproptosis-score groups, with the *x*-axis representing gene ratio, the *y*-axis showing pathway names, and color indicating adjusted *p*-values. (**H**) Bubble plot of KEGG pathway enrichment analysis, showing the top 20 signaling pathways enriched among the differentially expressed genes.

To evaluate the activation status of the cuproptosis pathway across different cell types, we calculated a cuproptosis pathway activity score for each cell using the AUCell algorithm, based on the 13 core cuproptosis-related genes defined by Tsvetkov et al. [11]. (*Fdx1*, *Lias*, *Lipt1*, *Dld*, *Dlat*, *Pdha1*, *Pdhb*, *Mtf1*, *Gls*, *Cdkn2a*, *Slc31a1*, *Atp7a*, *Atp7b*). UMAP overlay plots revealed pronounced regional enrichment of the cuproptosis pathway score in specific cell subpopulations, with acinar cells showing the highest scores, indicating selective pathway activation in this population (Figure 2A). Violin plots comparing the score distribution across cell types confirmed that acinar cells had significantly higher cuproptosis pathway scores than other cell types, while ductal cells, neutrophils, and macrophages exhibited relatively lower scores (Figure 2B). Bubble plots of the average expression of the 13 core cuproptosis genes across cell types showed higher abundance of most genes in acinar cells compared to other cell types (Figure 2C). Split bubble plots of expression changes in acinar cells by experimental group revealed generally higher expression of each gene in the AP group relative to the NFD group, indicative of coordinated upregulation of cuproptosis-related genes in acinar cells under acute pancreatitis conditions (Figure 2D). Heatmaps of the expression patterns of the 13 core cuproptosis genes across cell types (red, high expression; blue, low expression) displayed a distinct high-expression region in acinar cells, supporting the cell-type specificity of a cuproptosis-related signature (Figure 2E). Within the acinar cell population, all 13 cuproptosis genes were upregulated in the AP group compared to the NFD group, with eight reaching statistical significance (adjusted *p* < 0.05). The most prominently changed genes were *Gls* (log_2_FC = 4.15) and *Fdx1* (log_2_FC = 1.96). Additionally, *Pdha1*, *Dlat*, *Pdhb*, *Lias*, *Dld*, and *Slc31a1* were significantly upregulated. These genes span key steps including copper uptake (*Slc31a1*), effector molecules (*Fdx1*), target complexes (PDC subunits), and metabolic reprogramming (*Gls*), suggesting systemic upregulation of a cuproptosis-related program in acinar cells under AP conditions. Split violin plots of cuproptosis pathway scores in AP and NFD acinar cells showed significantly higher scores in the AP group, suggesting that acute pancreatitis is associated with selective upregulation of a cuproptosis-related signature in acinar cells (Figure 2F).

To explore the downstream biological functions of cuproptosis-related signaling pathways, we stratified acinar cells into high- and low-cuproptosis-score groups based on the median cuproptosis pathway score and performed enrichment analysis of differentially expressed genes. Gene Ontology (GO) biological process (BP) enrichment analysis showed that differentially expressed genes in the high-score group were significantly enriched in regulation of apoptotic signaling pathways, immune cell activation and differentiation, and regulation of nucleic acid and protein metabolism (including regulation of mRNA metabolic processes, regulation of RNA splicing, and protein complex assembly) (Figure 2G). Kyoto Encyclopedia of Genes and Genomes (KEGG) enrichment analysis further indicated that differentially expressed genes were mainly enriched in immune and inflammatory signaling pathways (PI3K-Akt, Toll-like receptor, chemokine, and C-type lectin receptor signaling) and in processes related to cytoskeletal and cell adhesion regulation (Figure 2H). In addition, exploratory hdWGCNA, transcription factor activity inference, and CellChat analyses were performed. Because these analyses are primarily inferential and hypothesis-generating, their detailed results and figures are provided in the Supplementary Results and Supplementary Materials Figures S1–S3, and they should not be interpreted as direct evidence of causality. Briefly, hdWGCNA identified modules correlated with AP status and the cuproptosis-related score; TF activity inference suggested SPI1 and AP-1-related factors as potential upstream regulators; and CellChat analysis suggested enhanced MIF/CCL/Annexin/Galectin signaling in AP. These findings require further experimental validation.

### 2.3. Acinar Cell Injury Trajectory and Temporal Dynamics of Cuproptosis

**Figure 3:**
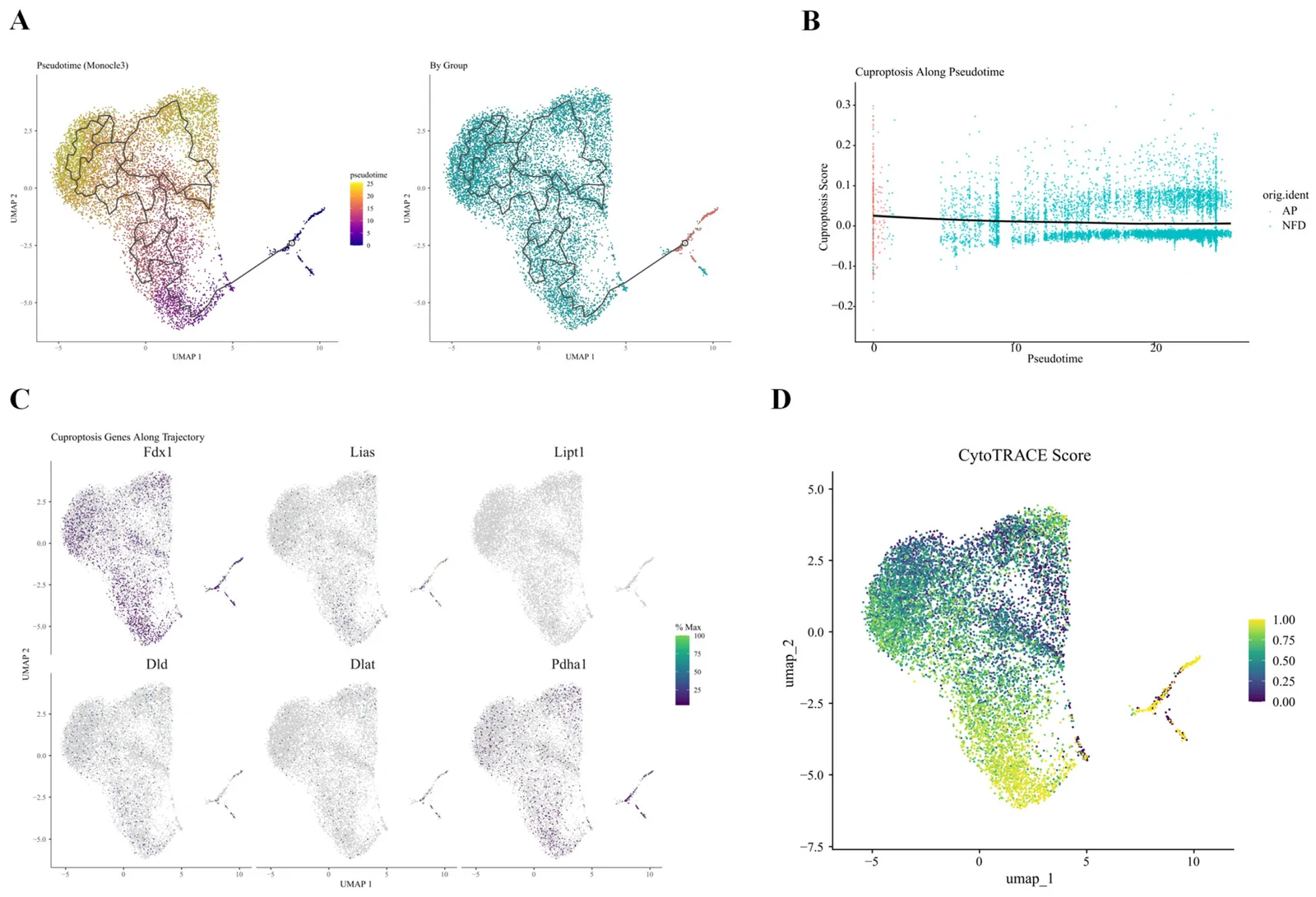
Temporal dynamics of cuproptosis along the acinar cell injury trajectory. (**A**) Two-panel UMAP plots showing the Monocle3-reconstructed pseudotime trajectory of acinar cells. The left panel is colored by pseudotime values, and the right panel is colored by sample groups. (**B**) Scatter plot overlaid with LOESS locally weighted regression curve showing the trend of cuproptosis pathway scores along pseudotime. The *x*-axis represents pseudotime values, the *y*-axis represents cuproptosis scores, the red curve indicates the fitted line, and the gray band denotes the 95% confidence interval. (**C**) Multi-panel UMAP plots displaying the expression distributions of representative core cuproptosis genes along the pseudotime trajectory, with color intensity reflecting gene expression levels. (**D**) UMAP plot overlaid with CytoTRACE stemness scores, with color intensity indicating the degree of differentiation.

To investigate the dynamic relationship between a cuproptosis-related signature and the progression of acinar cell injury, we extracted all acinar cells (*n* = 10,155) and constructed a pseudotime trajectory using Monocle3. Setting the centroids of control cells as the origin caused pseudotime values to increase progressively from the normal toward the injured state. In two-panel UMAP plots, the left panel colored by pseudotime showed a continuous transition of acinar cells from low to high values along the trajectory, while the right panel colored by sample group demonstrated that AP cells concentrated in high-pseudotime regions and NFD cells localized predominantly in low-pseudotime regions (Figure 3A). A LOESS locally weighted regression curve was fitted to visualize the trend of cuproptosis pathway scores across pseudotime. The scatter plot revealed that cuproptosis scores rose steadily with increasing pseudotime, and the narrow 95% confidence interval of the fit indicated high robustness of this trend (Figure 3B). This gradual upward pattern suggests that cuproptosis is not abruptly triggered at a single time point but instead intensifies in parallel with the stepwise aggravation of acinar cell injury. Multi-panel UMAP plots displayed the expression distributions of representative core cuproptosis genes along the pseudotime trajectory. These results showed marked upregulation of genes including *Gls*, *Fdx1*, *Pdha1*, *Dlat*, *Slc31a1*, and *Lias* in high-pseudotime (AP-enriched) regions, while their expression remained relatively low in low-pseudotime regions (Figure 3C). A spatial autocorrelation test (graph_test) confirmed that expression changes of these cuproptosis-related genes exhibited significant pseudotime dependence (*p* < 0.001), supporting an association between a cuproptosis-related transcriptional program and acinar cell injury progression. To further evaluate how the differentiation status of acinar cells varied along pseudotime, we computed individual cell stemness scores using the CytoTRACE algorithm. UMAP plots overlaid with CytoTRACE scores revealed that cells in the high-pseudotime region possessed higher stemness scores, indicating lower differentiation, whereas cells in the low-pseudotime region showed comparatively lower differentiation potential (Figure 3D). These findings suggest that, under AP conditions, acinar cells may concurrently undergo dedifferentiation or lineage reprogramming alongside progressive upregulation of a cuproptosis-related program.

### 2.4. Independent Validation Using a Single-Cell Dataset

**Figure 4:**
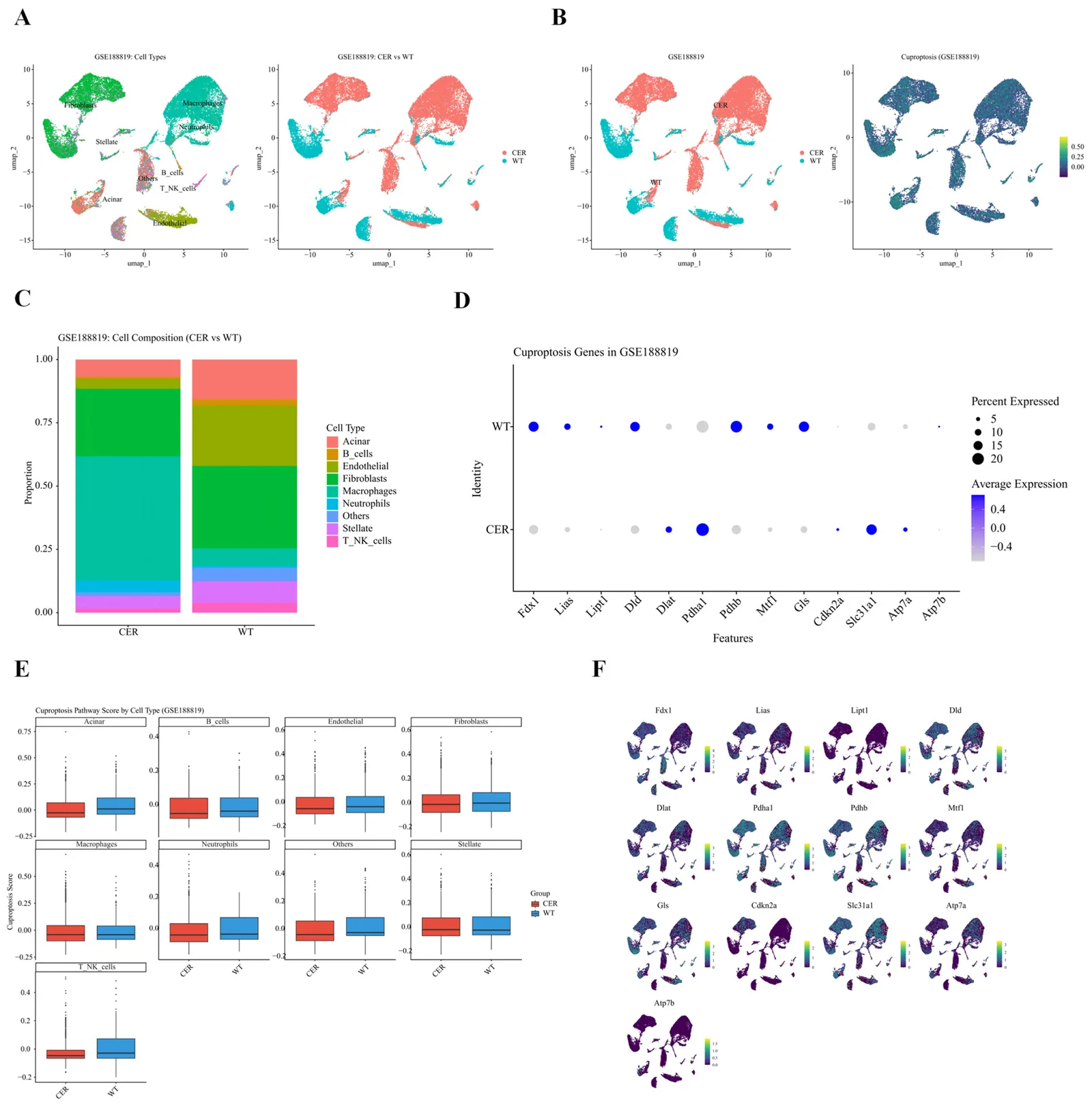
Independent validation using a single-cell dataset (GSE188819). (**A**) Two-panel UMAP plot showing cell-type annotations (left) and group distribution (CER caerulein-treated group vs. WT control group, right) in the independent mouse AP dataset GSE188819. (**B**) UMAP plot overlaid with cuproptosis pathway scores, with color intensity representing the score level. (**C**) Stacked bar plot showing the proportional distribution of cell types between the CER and WT control groups in the GSE188819 dataset. (**D**) Bubble plot comparing the expression differences of core cuproptosis genes between the CER and WT control groups in the GSE188819 dataset; dot size indicates the proportion of cells expressing the gene, and color depth represents the average expression level. (**E**) Box plots displaying the distribution of cuproptosis pathway scores stratified by cell type, with the horizontal axis representing cell types and the vertical axis representing pathway scores. The box represents the interquartile range (IQR), the horizontal line inside the box represents the median, the whiskers extend to 1.5 × IQR, and dots represent outliers. (**F**) UMAP plot overlaid with eigen-gene activity scores of each co-expression module, with different colors representing different modules, showing the enriched regions of each module in the UMAP space.

To validate the above core findings and rule out dataset-specific bias, we analyzed an independent single-cell dataset GSE188819 from a mouse acute pancreatitis model induced by caerulein, which comprised CER-treated and WT control groups. Two-panel UMAP plots showed the annotated cell types and group distributions; the left panel, colored by cell-type annotation, revealed a composition consistent with the primary dataset, while the right panel, colored by sample group, displayed the distribution of CER and WT cells in the UMAP space (Figure 4A).

UMAP plots overlaid with cuproptosis pathway scores revealed a pronounced dark-color enrichment in acinar cell regions in the CER group, whereas the WT controls showed overall lighter shading, indicating markedly elevated pathway activity under AP conditions (Figure 4B). Stacked bar plots of cell-type proportions between groups showed a marked increase in immune cells (neutrophils, macrophages, etc.) and a relative decrease in acinar cells in the CER group, consistent with the compositional shifts observed in the primary dataset (Figure 4C). Bubble plots comparing core cuproptosis gene expression between CER and WT groups showed that *Gls*, *Fdx1*, *Pdha1*, *Dlat*, *Slc31a1*, and *Lias* all exhibited upward trends in both the percentage of expressing cells and average expression level in acinar cells of the CER group (Figure 4D). Cell-type-stratified box plots of cuproptosis pathway scores indicated that in the CER group, the median score in acinar cells was significantly higher than in other cell types and showed the greatest elevation relative to WT controls (Figure 4E). UMAP plots colored by gene activity scores for each co-expression module revealed distinct spatial enrichment patterns, with the cuproptosis-related module displaying the highest activity in acinar cell regions. UMAP plots present core cuproptosis genes (*Gls*, *Fdx1*, *Pdha1*, *Dlat*), which each showed pronounced high-expression enrichment in acinar cell regions, further supporting the primary dataset finding that cuproptosis-related genes are specifically upregulated in acinar cells (Figure 4F). Collectively, cross-validation using the independent GSE188819 dataset supported the robustness and reproducibility of an acinar cell cuproptosis-related signature, ruling out any possibility that the core conclusions stemmed from dataset-specific bias in the primary dataset.

### 2.5. Exploratory Analysis Using a Human Whole-Blood Transcriptomic Dataset

**Figure 5:**
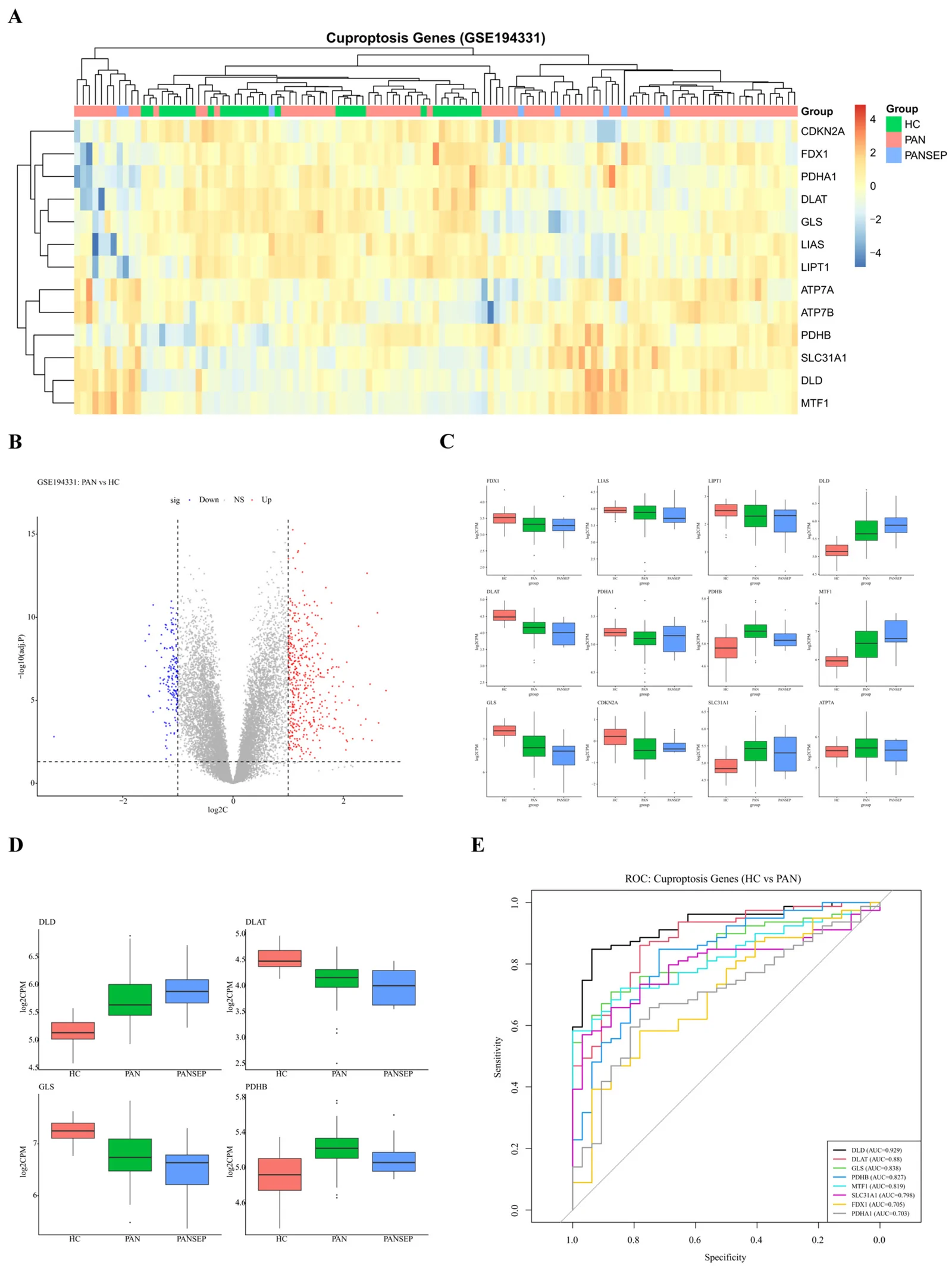
Exploratory analysis of cuproptosis-related genes in a human whole-blood transcriptomic dataset (GSE194331). (**A**) Heatmap displaying the expression patterns of the 13 core cuproptosis-related genes across 119 human samples, including healthy controls, acute pancreatitis, and acute pancreatitis with sepsis groups. Red indicates high expression, and blue indicates low expression. (**B**) Volcano plot showing genome-wide differential expression analysis results between acute pancreatitis patients and healthy controls in the GSE194331 dataset. Red dots represent significantly upregulated genes, blue dots represent significantly downregulated genes, and gray dots indicate genes with no significant difference. Dashed lines indicate the significance thresholds (|log2FC| > 1 and adjusted *p* < 0.05). (**C**) Box plots showing the expression level distribution of representative cuproptosis genes across the three human sample groups, with the horizontal axis representing group categories and the vertical axis representing gene expression levels. (**D**) Grouped box plots illustrating the expression differences of representative cuproptosis genes among the three groups, with the horizontal axis denoting group categories and the vertical axis denoting gene expression levels. The box represents the interquartile range (IQR), the horizontal line inside the box represents the median, whiskers extend to 1.5 × IQR, and dots represent outliers. (**E**) Receiver operating characteristic (ROC) curves showing an exploratory analysis of the potential discriminatory ability of cuproptosis genes in distinguishing acute pancreatitis patients from healthy controls.

To exploratorily evaluate the clinical relevance of cuproptosis-related genes in human acute pancreatitis, we analyzed the whole-blood transcriptomic dataset GSE194331, which included 32 healthy controls, 79 patients with acute pancreatitis, and eight patients with pancreatitis complicated by sepsis. The volcano plot depicted genome-wide differential expression between acute pancreatitis patients and healthy controls, with red dots marking significantly upregulated genes and blue dots marking significantly downregulated genes. Analysis revealed that multiple cuproptosis-related genes, including GLS, FDX1, and SLC31A1, displayed significant expression alterations in the peripheral blood of acute pancreatitis patients, with certain genes trending upward (Figure 5B). The heatmap of the 13 core cuproptosis genes across all 119 human samples illustrated expression patterns, where red denoted high expression and blue denoted low expression. In the acute pancreatitis group, multiple cuproptosis genes showed enrichment of high-expression (red) signals, whereas healthy controls predominantly exhibited low-expression (blue) patterns. The expression pattern in the pancreatitis with sepsis group resembled that of the acute pancreatitis group, but with even higher levels for some genes, suggesting a coordinated upregulation signature of cuproptosis genes under disease conditions (Figure 5A). Box plots of representative cuproptosis genes illustrated expression-level distributions across the three sample groups: healthy controls, acute pancreatitis, and pancreatitis with sepsis (Figure 5C). Grouped box plots comparing the three groups revealed that the expression levels of genes such as *GLS*, *FDX1*, *DLAT*, and *SLC31A1* increased stepwise from healthy controls to acute pancreatitis to pancreatitis with sepsis, indicating a progressive rise with disease severity (Figure 5D).

This exploratory gradient pattern suggested that cuproptosis-related genes might be associated with disease severity, although this may partly reflect changes in circulating immune-cell populations. We exploratorily assessed the potential ability of cuproptosis-related genes to discriminate acute pancreatitis patients from healthy controls using receiver operating characteristic (ROC) curve analysis. These exploratory curves suggested that several cuproptosis-related genes showed potential discriminatory signals with relatively high area under the curve (AUC) values: DLD (0.819), SLC31A1 (0.798), FDX1 (0.705), and PDHA1 (0.703), with the highest AUC (0.929), followed by DLAT (0.888), GLS (0.838), and PDHB (0.827) (Figure 5E). However, because these analyses were based on whole-blood transcriptomes and may partly reflect circulating immune-cell composition, they should be considered hypothesis-generating and not as evidence of established diagnostic utility.

### 2.6. Phenotypic Validation of Cuproptosis and Potential Ameliorative Effects of TTM in a Mouse Model of Severe Acute Pancreatitis

**Figure 6:**
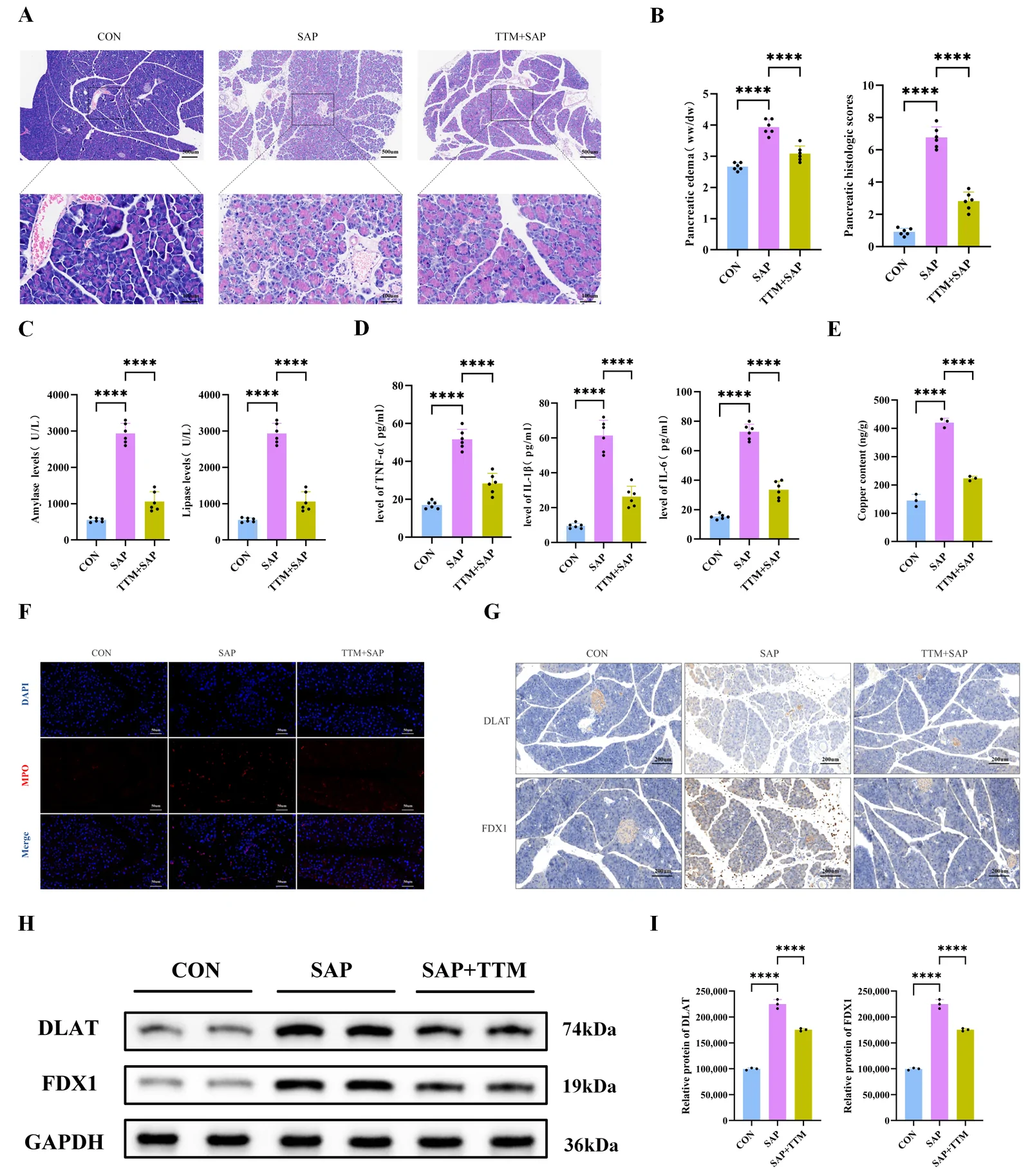
Phenotypic validation of cuproptosis and effects of TTM intervention in a mouse model of severe acute pancreatitis. (**A**) Hematoxylin and eosin (H&E) staining showing the histopathological morphology of pancreatic tissues in each group. Scale bars: 500 um for low-magnification images and 100 um for high-magnification images. (**B**) Bar graphs showing quantitative comparisons of histopathological scores and tissue edema among groups, with the horizontal axis representing groups and the vertical axis representing score values. (**C**) Bar graphs showing serum amylase and lipase activity levels in each group, with the horizontal axis representing groups and the vertical axis representing enzyme activity units. (**D**) Bar graphs showing serum concentrations of inflammatory cytokines (TNF-α, IL-1β, and IL-6) in each group, with the horizontal axis representing groups and the vertical axis representing cytokine concentrations. (**E**) Inductively coupled plasma mass spectrometry (ICP-MS) showing quantitative comparisons of total copper content in pancreatic tissues of each group, with the horizontal axis representing groups and the vertical axis representing copper content. (**F**) Representative immunofluorescence images of myeloperoxidase (MPO) in pancreatic tissues of each group. Scale bar: 50 um. (**G**) Representative immunohistochemical staining images of FDX1 and DLAT in pancreatic tissues of each group. Scale bar: 200 um. (**H**) Western blotting showing protein expression bands of ferredoxin 1 (FDX1) and dihydrolipoamide S-acetyltransferase (DLAT) in pancreatic tissues of each group. (**I**) Bar graphs showing quantitative comparisons of FDX1 and DLAT protein expression in pancreatic tissues among groups, with the horizontal axis representing groups and the vertical axis representing relative protein expression levels. Asterisks indicate statistical significance: **** *p* < 0.0001.

To further assess the cuproptosis-related signals identified by single-cell sequencing at the organismal level, we established a caerulein-induced severe acute pancreatitis (SAP) mouse model and administered the copper chelator TTM as an intervention. Hematoxylin and eosin staining revealed that pancreatic tissues from the SAP group exhibited marked interstitial edema, inflammatory cell infiltration, and disruption of acinar cell architecture, all of which were ameliorated by TTM intervention (Figure 6A). Histopathological scoring and quantitative analysis of edema further confirmed the attenuating effect of TTM on pancreatic tissue injury (Figure 6B). Along with the histological damage, serum amylase and lipase activities were markedly elevated in the SAP group, whereas TTM treatment significantly reduced both enzyme levels (Figure 6C). Serum cytokine measurements showed that TNF-α, IL-1β, and IL-6 levels were substantially increased in the SAP group, and TTM intervention led to decreases in inflammatory factors (Figure 6D). Inductively coupled plasma mass spectrometry (ICP-MS) measurements showed that the total copper content in pancreatic tissues was higher in the SAP group compared with the control group, and TTM treatment reduced pancreatic copper levels (Figure 6E). Copper ions are the essential upstream trigger for cuproptosis; the substantial elevation of copper content in SAP pancreatic tissues provides a potential material basis for cuproptosis-related cell death, while the copper-chelating action of TTM blocks this process at its source. MPO immunofluorescence staining of pancreatic tissues demonstrated a pronounced increase in neutrophil infiltration in the SAP group, while the intensity of MPO-positive signals was diminished in the TTM-treated group (Figure 6F). Collectively, these results indicate that the SAP mouse model was successfully established, and that TTM could alleviate pancreatic tissue injury and systemic inflammatory responses. Immunohistochemical staining further corroborated this trend at the tissue level, with enhanced immunoreactive signals for FDX1 and DLAT in the SAP group, whereas the staining intensity was decreased in the TTM group (Figure 6G).

Immunoblotting analysis revealed that the protein expression levels of FDX1 and DLAT were significantly upregulated in the pancreatic tissues of the SAP group, and both were reduced following TTM intervention (Figure 6H). Quantitative analysis of FDX1 and DLAT protein expression confirmed FDX1 and DLAT were significantly upregulated in the SAP group and reduced after TTM intervention (Figure 6I). FDX1 and DLAT are core effector molecules of the cuproptosis pathway; their coordinated upregulation in SAP pancreatic tissues suggests activation of a cuproptosis-related molecular program in the SAP model, and that TTM intervention attenuated this molecular event. Taken together, these histopathological, serological, immunological, and molecular biological lines of evidence support the presence of copper overload and a cuproptosis-related phenotype in the pancreatic tissues of SAP mice, and suggest that the copper chelator TTM may intervene in this pathological process.

### 2.7. In Vitro Phenotypic Validation of Cuproptosis in Acinar Cells and TTM Rescue Effects

**Figure 7:**
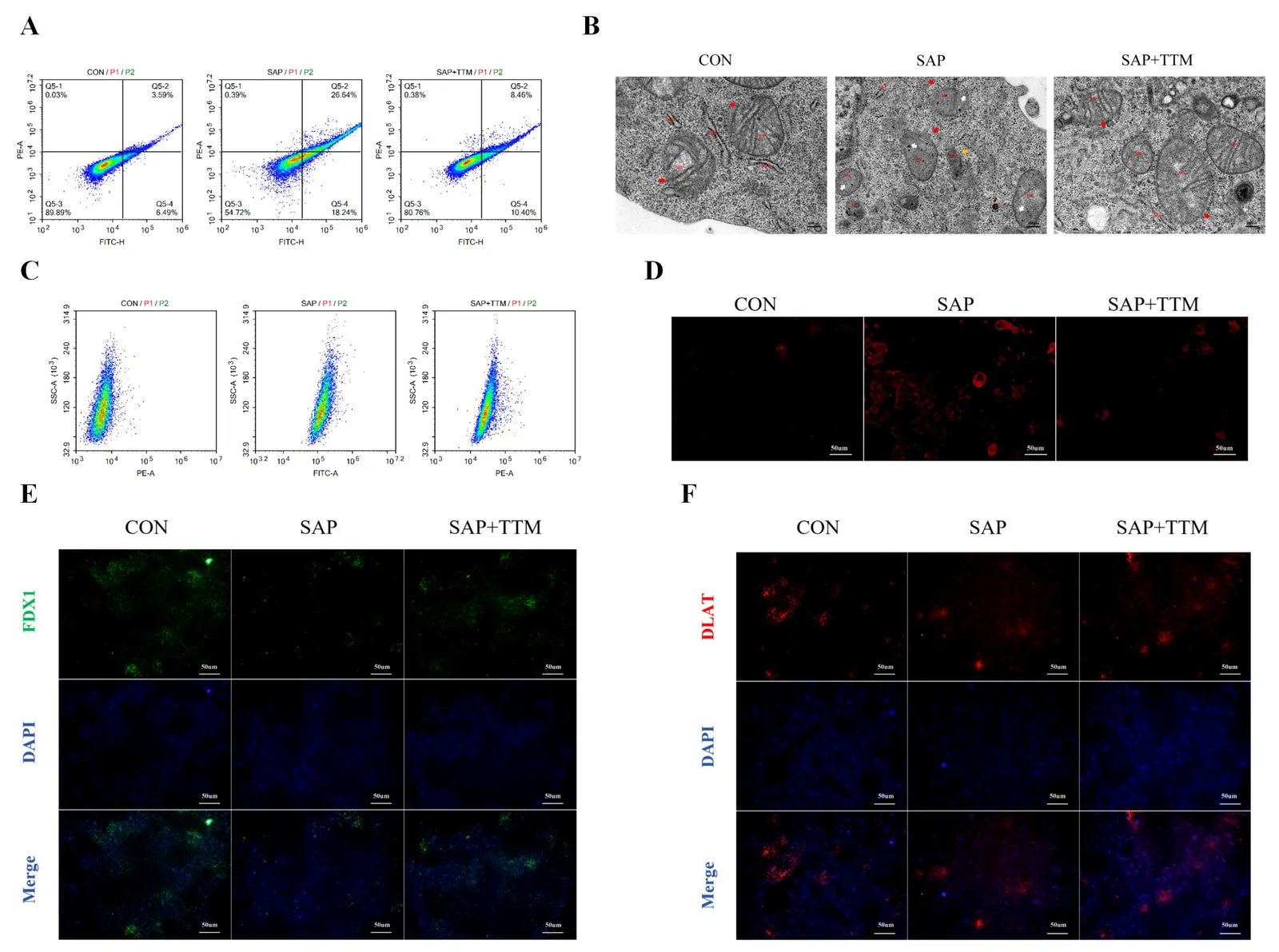
In vitro phenotypic validation of acinar cell cuproptosis and the rescue effect of TTM. (**A**) Flow cytometric dot plots show the apoptosis rates of acinar cells in each group, with groups on the horizontal axis and the percentage of apoptotic cells on the vertical axis. The color gradient indicates cell density, with blue representing low density and red representing high density. (**B**) Transmission electron microscopy images showing mitochondrial ultrastructure in pancreatic acinar cells from each group. Mit, mitochondria; RER, rough endoplasmic reticulum. Red arrows indicate discontinuous mitochondrial membranes or loss of ribosomes from the RER surface; yellow arrows indicate shrunken mitochondria with increased membrane density; white arrows indicate electron-dense granules in the mitochondrial matrix. Scale bar: 500 nm. (**C**) Flow cytometric dot plots show intracellular copper ion fluorescence intensity in acinar cells of each group, with groups on the horizontal axis and mean fluorescence intensity on the vertical axis. The color gradient indicates cell density, with blue representing low density and red representing high density. (**D**) Immunofluorescence staining presents representative images of intracellular copper accumulation in each group. Scale bar: 50 um. (**E**) Immunofluorescence staining shows the expression distribution of ferredoxin 1 (FDX1) in acinar cells per group. Scale bar: 50 um. (**F**) Immunofluorescence staining shows the expression distribution of dihydrolipoamide S-acetyltransferase(DLAT) in each group. Scale bar: 50 um.

To further assess whether a cuproptosis-related phenotype occurs at the acinar cell level while excluding confounding systemic inflammatory responses present in vivo, we used 266-6 mouse pancreatic acinar cells, established a caerulein-induced injury model, and treated the cells with TTM. Flow cytometric analysis revealed that the apoptosis rate in the SAP group was higher than that in the control group, and TTM intervention reduced this rate, indicating a protective effect on acinar cells (Figure 7A). Transmission electron microscopy showed normal mitochondrial morphology in control acinar cells, with well-preserved, clearly defined cristae arranged in a lamellar pattern and distinct bilayer membrane boundaries. In contrast, acinar cells in the SAP group exhibited typical cuproptosis-associated ultrastructural changes: mitochondria were shrunken and smaller, with most cristae absent or disrupted, increased matrix electron density, blurred bilayer membranes, and some showing vacuolar degeneration. Notably, in the SAP + TTM group, TTM intervention attenuated these mitochondrial abnormalities (Figure 7B). These morphological features were highly consistent with the cuproptosis-related mitochondrial damage phenotype reported by Tsvetkov et al. [11]. We measured intracellular copper accumulation in acinar cells using flow cytometry and immunofluorescence staining. Flow cytometry demonstrated that copper fluorescence intensity in SAP acinar cells was higher than in control cells, and TTM treatment reduced this signal (Figure 7C). Immunofluorescence staining confirmed this enrichment at the single-cell level, revealing enhanced copper fluorescence in SAP cells that was attenuated after TTM treatment (Figure 7D). Copper ions are the critical upstream initiators of cuproptosis; the accumulation of copper in SAP acinar cells therefore supplies the requisite trigger for the pathway, and the copper-chelating action of TTM blocks this initiation step at the cellular level. Immunofluorescence staining was used to assess the expression of the core cuproptosis regulatory proteins FDX1 and DLAT in acinar cells. FDX1 fluorescence signals were stronger in SAP acinar cells than in control cells, and TTM intervention reduced FDX1 expression (Figure 7E).

DLAT expression exhibited a similar trend, with enhanced fluorescence in the SAP group and decrease after TTM treatment (Figure 7F). FDX1 and DLAT are core executioners in the cuproptosis pathway. Their coordinated upregulation in SAP acinar cells further supports activation of a cuproptosis-related program at the cellular level. In summary, based on apoptosis assays, ultrastructural analysis, copper accumulation measurements, and cuproptosis marker expression, our data suggest that caerulein induces a cuproptosis-related phenotype in acinar cells and that TTM ameliorates this cell death phenotype at the cellular level, providing a cellular-level mechanistic rationale for the potential protective effects of TTM observed in vivo.

### 2.8. In Vitro Phenotypic Validation of Cuproptosis-Related Mitochondrial Dysfunction and Oxidative Stress and TTM Rescue Effects

**Figure 8:**
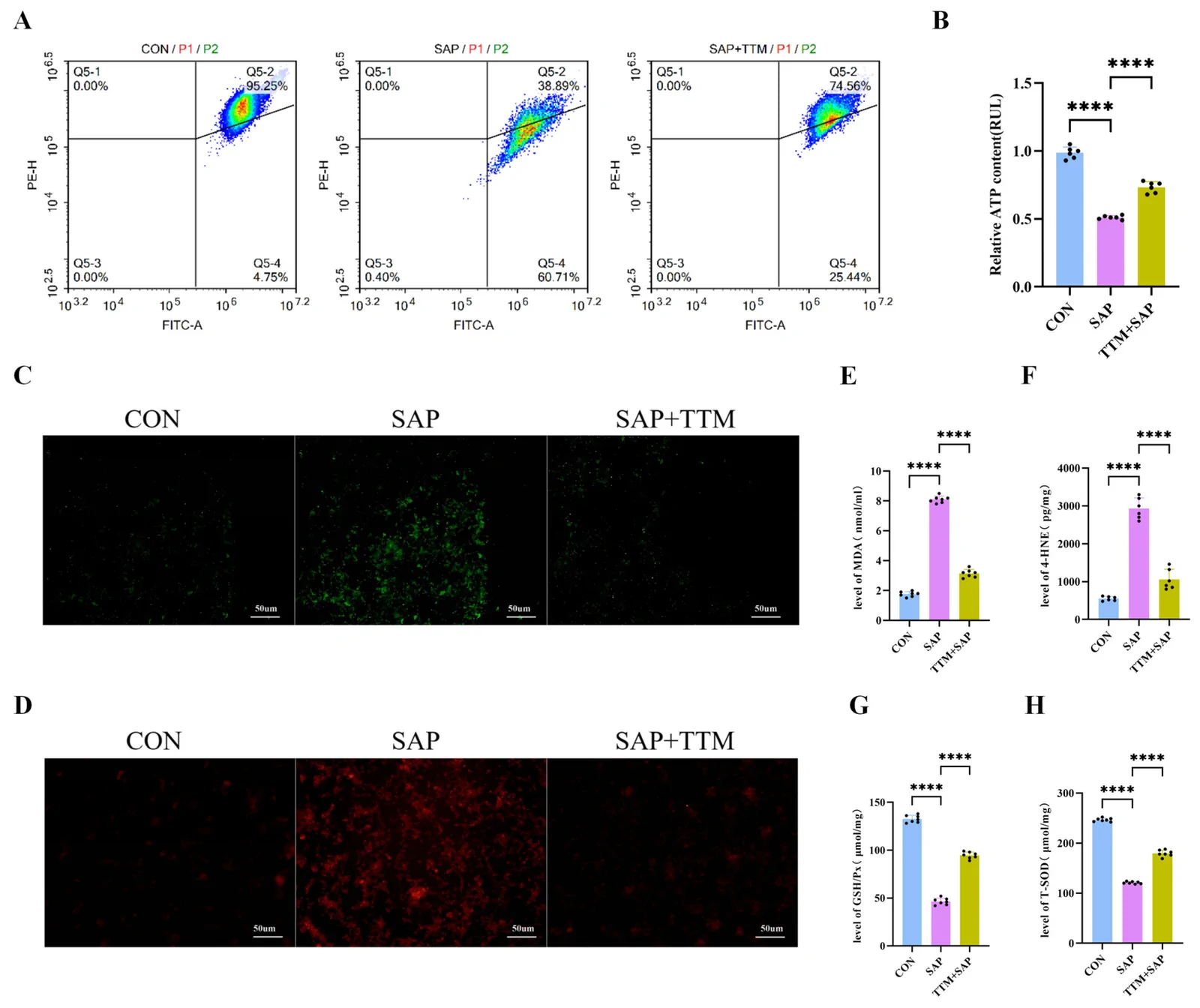
In vitro phenotypic validation of cuproptosis-related mitochondrial dysfunction and oxidative stress, and the rescuing effect of TTM. (**A**) Flow cytometry scatter plots showing mitochondrial membrane potential changes in acinar cells from each group detected by JC-1 probe. The *x*-axis represents FITC-A and the *y*-axis represents PE-H, which indicate damaged and normal mitochondrial membrane potential, respectively. The color gradient indicates cell density, with blue representing low density and red representing high density. (**B**) Bar graph showing relative ATP content in acinar cells from each group measured by chemiluminescence assay. The *x*-axis represents groups and the *y*-axis represents relative ATP levels. (**C**) Fluorescence images and quantitative bar graphs showing total ROS levels in acinar cells from each group detected by DCFH-DA probe. The *x*-axis represents groups and the *y*-axis represents relative fluorescence intensity. Scale bar: 50 um. (**D**) Fluorescence images and quantitative bar graphs showing mitochondrial ROS levels in acinar cells from each group detected by MitoSOX probe. The *x*-axis represents groups and the *y*-axis represents relative fluorescence intensity. Scale bar: 50 um. (**E**) Bar graph showing quantitative comparison of malondialdehyde (MDA) content in acinar cells from each group. The *x*-axis represents groups and the *y*-axis represents MDA content. (**F**) Bar graph showing quantitative comparison of 4-hydroxynonenal (4-HNE) levels in acinar cells from each group. The *x*-axis represents groups and the *y*-axis represents 4-HNE levels. (**G**) Bar graph showing quantitative comparison of reduced glutathione/oxidized glutathione (GSH/GSSG) ratio in acinar cells from each group. The *x*-axis represents groups and the *y*-axis represents GSH/GSSG ratio. (**H**) Bar graph showing quantitative comparison of total superoxide dismutase (T-SOD) activity in acinar cells from each group. The *x*-axis represents groups and the *y*-axis represents T-SOD activity units. Data are presented as mean ± SD. **** indicates *p* < 0.0001.

To determine how activation of a cuproptosis-related program alters mitochondrial function and redox status in acinar cells, we measured mitochondrial membrane potential, ATP content, ROS levels, and antioxidant defense parameters in each group. JC-1 flow cytometry showed a significant decline in mitochondrial membrane potential in SAP acinar cells, indicated by a smaller proportion of PE-H-positive cells and a larger proportion of FITC-A-positive cells; TTM treatment improved this potential (Figure 8A).

Aligned with the loss of mitochondrial membrane potential, ATP levels in SAP acinar cells were lower than in controls, and TTM treatment increased them (Figure 8B). Mitochondrial membrane potential is the principal driver of ATP synthesis via oxidative phosphorylation; the synchronous decline of both parameters in SAP acinar cells suggests that a cuproptosis-related process causes profound mitochondrial bioenergetic failure, and that TTM-mediated copper chelation preserves mitochondrial energy production. We next examined oxidative stress in these cells. DCFH-DA staining showed that total ROS levels were higher in SAP acinar cells than in controls, and TTM reduced total ROS (Figure 8C). MitoSOX detection of mitochondrial superoxide revealed elevated mitochondrial ROS in the SAP group, which were attenuated by TTM (Figure 8D). Mitochondria represent the primary source of intracellular ROS; cuproptosis-induced mitochondrial injury invariably causes electron leakage from the respiratory chain, spurring a burst of mtROS. The coordinated rise in total ROS and mtROS points to mitochondria as the main origin of oxidative stress in SAP cells, and demonstrates that TTM curbs excessive ROS production at its source by preserving mitochondrial integrity. Measurement of lipid peroxidation end-products revealed higher levels of MDA and 4-HNE in SAP acinar cells compared to controls, and TTM lowered both (Figure 8E,F). MDA and 4-HNE are terminal breakdown products of ROS-attacked polyunsaturated fatty acids; their accumulation indicates the extent of oxidative damage to cell membranes, providing further support for cuproptosis-related oxidative injury. Evaluation of the antioxidant system revealed that the ratio of reduced to oxidized glutathione (GSH/GSSG) was lower in SAP acinar cells than in controls, and TTM treatment restored this ratio (Figure 8G). Total superoxide dismutase (T-SOD) activity was lower in the SAP group than in controls, and TTM treatment rescued SOD activity (Figure 8H). The GSH/GSSG ratio is a key metric of intracellular redox status; its decline signals that copper overload depletes the reduced glutathione pool. SOD serves as the first line of defense against superoxide; diminished SOD activity further amplifies oxidative damage. TTM improved the cellular antioxidant defense by curtailing the ongoing copper influx. Integrating the evidence across mitochondrial membrane potential, ATP synthesis, ROS burst, lipid peroxidation, and antioxidant exhaustion, we conclude that SAP acinar cells undergo mitochondrial dysfunction and oxidative injury upon activation of a cuproptosis-related program, with the two processes reinforcing each other in a vicious cycle. By chelating copper ions and preventing copper-mediated initiation of a cuproptosis-related process, TTM may simultaneously preserve mitochondrial functional integrity and attenuate the cascade amplification of oxidative stress at its source.

## 3. Discussion

Since cuproptosis was first defined by Tsvetkov et al. [11], it has attracted considerable interest across fields such as oncology, neurodegenerative diseases, and ischemia–reperfusion injury [23,24,25]. Cuproptosis, a unique programmed cell death modality, depends on the direct binding of copper ions to lipoylated enzymes of the tricarboxylic acid (TCA) cycle; its execution is closely associated with mitochondrial metabolic status and intracellular redox balance [26]. Recent studies suggest a bidirectional interplay between copper metabolism and inflammatory responses: inflammatory signals can promote copper influx by upregulating copper transporter expression, while copper ions themselves can activate pro-inflammatory signaling pathways and induce oxidative stress. This interaction has received preliminary validation in animal models of infectious and metabolic inflammation [27,28]. The pathological hallmarks of acute pancreatitis include aberrant intra-acinar trypsinogen activation, a pronounced local and systemic inflammatory storm, and synergistic activation of oxidative and endoplasmic reticulum (ER) stress; however, whether these changes are linked to disturbances in copper metabolism that trigger cuproptosis remains unclear [29,30,31]. Prior studies on acinar cell death in acute pancreatitis have concentrated on necrosis, apoptosis, pyroptosis, and ferroptosis [32]. The present study is the first to report an association between a cuproptosis-related signature and acinar cell injury in acute pancreatitis at single-cell resolution, systematically revealing the selective upregulation of a cuproptosis-related program in acinar cells and its dynamic evolution during disease progression. In vitro and in vivo experiments provide evidence consistent with the involvement of copper accumulation and coordinated upregulation of FDX1 and DLAT proteins in pancreatitis-associated acinar cell injury. These findings address a gap in knowledge concerning cuproptosis in acute pancreatitis and provide new experimental evidence for understanding the molecular basis of acinar cell injury within the inflammatory microenvironment.

Upon entering cells, copper ions are imported via SLC31A1. Inside the cell, Cu^2+^ is reduced to Cu^+^ by FDX1; the Cu^+^ then binds to lipoacylated DLAT in the tricarboxylic acid (TCA) cycle, inducing aberrant protein oligomerization and loss of iron–sulfur cluster proteins [33]. This reductive activation represents a critical rate-limiting step in the initiation of cuproptosis. In the present study, severe acute pancreatitis (SAP) acinar cells exhibited elevated copper content and synchronous upregulation of FDX1 and DLAT protein levels. FDX1, the enzyme that reductively activates copper, may be upregulated in response to the elevated copper load, reflecting a compensatory enhancement that accelerates copper reduction [34]. The upregulation of DLAT, a direct binding target of copper ions, may expand the pool of lipoacylated substrates available for copper attack [35]. The concurrent upregulation of both components under copper overload suggests that, during acute inflammation, acinar cells may upregulate key elements of the cuproptosis pathway to cope with copper stress; paradoxically, this compensatory response renders them more susceptible to cuproptosis, establishing a potential pathological positive feedback loop. Clear experimental evidence indicates that excessive copper accumulation damages mitochondrial ultrastructure. Cu^+^ binding to lipoacylated DLAT induces aberrant protein oligomerization, which disrupts proteostasis in the mitochondrial matrix and subsequently leads to progressive destruction of the inner mitochondrial membrane and cristae [36]. Ex vivo studies have consistently shown that copper ions cause mitochondrial cristae fracture, matrix dissolution, and loss of membrane integrity [37]. The mitochondrial shrinkage, cristae disappearance, and blurred outer membrane observed in SAP acinar cells in this study are consistent with these reported morphological features. Given that the copper ions in this study originated from endogenous accumulation rather than exogenous supplementation, this morphological congruence indicates that the final pattern of mitochondrial structural damage is similar irrespective of how copper ions enter the mitochondrial microenvironment.

Previous studies have established an association between cuproptosis activation and disease severity [38]. Zhang et al. developed a prognostic risk model for lung adenocarcinoma based on cuproptosis- and immune-related genes and demonstrated that patients in the high-risk group had significantly lower survival rates, reduced immune infiltration, and lower immune checkpoint expression than those in the low-risk group [39]. Chong et al. constructed a cuproptosis signature risk scoring system for gastric cancer using transcriptomic data from 16 cuproptosis-related genes and found that patients in the low-risk-score group not only achieved significantly superior overall survival but also exhibited a higher tumor mutational burden than the high-risk group [40]. However, the association between cuproptosis and the progression of acute pancreatitis has not been previously reported. In this study, pseudotime analysis revealed a monotonically increasing cuproptosis score as acinar cells transitioned from a normal to an injured state. Meanwhile, data from a human clinical cohort showed a stepwise elevation in the expression levels of cuproptosis-related genes across the three groups: healthy controls, acute pancreatitis, and pancreatitis complicated by sepsis. These two independent lines of evidence—one derived from single-cell dynamic tracing and the other from cross-sectional comparisons of population samples—collectively suggest that the activation level of a cuproptosis-related program is associated with the severity of acute pancreatitis. This finding suggests that cuproptosis-related processes are not merely bystanders in the disease process but may contribute to the progression of acinar cell injury.

The reciprocal regulation between inflammation and copper metabolism has been established in multiple experimental systems. TNF-α upregulates SLC31A1 expression and promotes copper influx in various cell types [18]; IL-1β and IL-6 have also been shown to modulate copper transporter transcription via the STAT3/NF-κB pathway [41]. Conversely, elevated intracellular copper levels can activate the NLRP3 inflammasome and the MAPK pathway, thereby exacerbating the release of pro-inflammatory cytokines [42]. However, these studies have primarily focused on macrophages and tumor cells, and whether inflammatory signals are associated with the transcriptional upregulation of cuproptosis-related genes in acinar cells remains unexplored. In the present study, we observed a simultaneous increase in inflammatory cytokines and upregulation of key cuproptosis genes in caerulein-stimulated acinar cells. Based on these findings, exploratory transcription factor activity inference suggested SPI1, FOS, and JUN as potential upstream regulators of core cuproptosis-related genes. SPI1, a master transcription factor for myeloid cell differentiation and inflammatory responses, directly binds to PU.1-binding motifs in the promoters of various pro-inflammatory genes [43]. In macrophages, SPI1 drives the transcription of pro-inflammatory cytokines upon stimulation with TNF-α and TLR ligands [44]. In our study, SPI1 activity was elevated under AP conditions, and exploratory cell–cell communication analysis suggested enhanced inflammatory signaling pathways between SPI1-high myeloid cells and acinar cells, raising the possibility that SPI1 may act as an intermediary linking inflammatory signals to transcriptional responses in acinar cells. FOS and JUN form the AP-1 complex, a classical downstream effector of TNF-α and IL-1β, and induce target gene transcription in response to MAPK and JNK pathway activation in various cell types [45]. The AP-1 complex has been implicated in regulating stress- and inflammation-related genes in pancreatic acinar cells [46]. Collectively, the concurrence of elevated inflammatory cytokines and cuproptosis-related activation, the altered transcription factor activities, and the documented roles of SPI1 and AP-1 in inflammatory transcriptional regulation lead us to propose that the inflammatory microenvironment may be associated with a transcriptional milieu permissive for initiation of a cuproptosis-related program in acinar cells, potentially mediated by transcription factors such as SPI1 and AP-1. This model is speculative and requires further validation. SPI1 may potentially act through binding to cuproptosis-related gene promoters, whereas AP-1 may contribute via inflammatory signals through the MAPK/JNK pathway; these possibilities require experimental validation.

Previous studies have repeatedly confirmed the association between cuproptosis and mitochondrial dysfunction. Copper ions attack lipoylated enzymes in the tricarboxylic acid (TCA) cycle, directly impairing the function of respiratory chain complexes and disrupting electron transfer [47]. When the respiratory chain is damaged, obstructed electron flow diminishes the proton-motive force, leaving ATP synthase unable to maintain normal ATP output. Simultaneously, electrons stalled at complexes I and III of the electron transport chain readily reduce molecular oxygen to superoxide, a major source of mitochondrial ROS [48]. Copper-driven disruption of the TCA cycle also lowers the yield of NADH and FADH_2_, further restricting electron supply and perpetuating respiratory chain impairment [49]. These mechanistic links have been substantiated in multiple cell types, and the concurrent ATP depletion, mitochondrial membrane potential collapse, and mtROS burst observed in this study align well with this pathway. The reciprocal amplification of mitochondrial dysfunction and oxidative stress characterizes disease progression. Respiratory chain-derived ROS can directly oxidize aconitase—the iron–sulfur cluster enzyme of the TCA cycle—thereby further diminishing TCA cycle flux [50]. Concomitantly, ROS-mediated peroxidation of mitochondrial membrane lipids increases inner membrane permeability and proton leak, further compromising oxidative phosphorylation efficiency. With respect to antioxidant defenses, GSH serves as the predominant intracellular copper chelator and ROS scavenger, and its regeneration depends heavily on NADPH. During a massive copper influx, GSH is directly depleted by copper chelation; meanwhile, mitochondrial ROS continually drive its oxidation to GSSG. This combined assault of copper-induced depletion and ROS-driven oxidation sharply lowers the GSH/GSSG ratio, overwhelming cellular compensatory capacity and shifting oxidative damage from a manageable to an uncontrollable state [51,52]. As the central hub of energy metabolism in acinar cells, mitochondrial dysfunction directly compromises cell survival and functional maintenance. Our data suggest that cuproptosis-related mitochondrial dysfunction not only results in impaired ATP production and an oxidative burst, but may also exacerbate acinar cell injury by disrupting energy-dependent homeostatic mechanisms. Moreover, oxidative stress arising from mitochondrial dysfunction further compromises membrane integrity and activates local inflammatory responses, thereby propelling the progression of acute pancreatitis from initial acinar cell damage to a systemic inflammatory storm.

These findings provide a theoretical basis for further investigating cuproptosis as a potential therapeutic target in acute pancreatitis. Copper levels were reduced, the aberrant upregulation of FDX1 and DLAT was attenuated, and pancreatic injury was ameliorated by TTM both in vitro and in vivo, suggesting that targeting copper accumulation may be a potential therapeutic strategy. Furthermore, in a human whole-blood transcriptomic cohort, a gradient association was observed between cuproptosis-related gene expression and disease severity, and exploratory ROC analysis suggested that multiple genes showed potential discriminatory signals, with relatively high AUC values. However, because this dataset consists of whole blood rather than pancreatic tissue, these changes may partly reflect alterations in circulating immune-cell populations, and the potential clinical predictive value of these genes remains exploratory and requires further validation. In summary, through single-cell profiling, transcriptional regulatory inference, and in vitro and in vivo phenotypic validation, this study identifies a cuproptosis-related phenotype in acute pancreatitis and reveals the selective activation features of a cuproptosis-related program in acinar cells and its potential association with the inflammatory microenvironment. The major limitations of this study include the following: the evidence for cuproptosis in acinar cells remains largely phenotypic and, although consistent with a cuproptosis-related phenotype, is not specific to cuproptosis; direct causal proof of this cell-death mechanism has not yet been provided; and the specific regulatory networks of FDX1 and DLAT under copper overload conditions, as well as the causal relationship between cuproptosis and the inflammatory response, remain to be further clarified. In addition, the CellChat and transcription factor analyses are exploratory and hypothesis-generating, and although they suggest potential links between inflammatory signaling and a cuproptosis-related program, they cannot establish causality and require further experimental validation. In addition, we did not measure mitochondrial DNA copy number; therefore, we cannot formally exclude a contribution of altered mitochondrial quantity. However, the ultrastructural and functional data suggest that the observed mitochondrial defects primarily reflect functional compromise rather than a reduction in mitochondrial number. Future studies should investigate the mechanisms of cuproptosis in acinar cells during acute pancreatitis, with a particular focus on the transcriptional regulatory mechanisms by which copper ion accumulation triggers FDX1 and DLAT and the crosstalk between mitochondrial dysfunction and inflammatory signaling.

## 4. Materials and Methods

### 4.1. Data Acquisition and Processing

The mouse single-cell RNA-sequencing datasets GSE279876 and GSE188819, and the human whole-blood transcriptome dataset GSE194331, were downloaded from the Gene Expression Omnibus (GEO) database (https://www.ncbi.nlm.nih.gov/geo/ (accessed on 10 April 2026)). GSE279876 contained pancreatic tissue samples from four groups: NFD, AP, HFD, and HAP. GSE188819 contained two groups: WT and caerulein-treated. GSE194331 included 32 healthy controls, 79 acute pancreatitis patients, and 8 pancreatitis patients with sepsis. Quality control was performed using Seurat v5 with the following criteria: each gene expressed in at least 10 cells; each cell expressing 300–5000 genes; total UMI counts per cell > 500; and mitochondrial gene content < 10%. A total of 36,794 high-quality cells were retained. All analyses were performed in R 4.1.1.

### 4.2. Single-Cell Atlas Construction and Cell-Type Annotation

Single-cell data were normalized using the LogNormalize method in Seurat v5. Principal component analysis was performed based on the top 2000 highly variable genes, and the first 30 principal components were used for UMAP dimensionality reduction and clustering (resolution = 0.5). Automated cell-type annotation was performed using the SingleR package with the MouseRNAseqData reference database, followed by manual curation using canonical marker genes (*Prss1*/*Cpa1* for acinar cells, *S100a8*/*S100a9* for neutrophils, *Cd68*/*Cd163* for macrophages, *Krt19*/*Sox9* for ductal cells). Ten distinct cell types were ultimately defined. Batch effects across experimental groups were corrected using the Harmony algorithm.

### 4.3. Cuproptosis Pathway Scoring

A set of 13 core cuproptosis-related genes (*Fdx1*, *Lias*, *Lipt1*, *Dld*, *Dlat*, *Pdha1*, *Pdhb*, *Mtf1*, *Gls*, *Cdkn2a*, *Slc31a1*, *Atp7a*, *Atp7b*) was retrieved from the previous literature [11] Cuproptosis pathway activity scores were calculated for each cell using two independent methods: the AddModuleScore function in Seurat and the AUCell algorithm. The two scoring results were cross-validated in the UMAP space.

### 4.4. Exploratory Co-Expression, Transcription Factor, and Cell–Cell Communication Analyses

High-dimensional weighted gene co-expression network analysis (hdWGCNA), transcription factor (TF) activity inference, and CellChat analysis were performed as exploratory analyses. Detailed parameters are provided in the Supplementary Methods. In brief, hdWGCNA was performed using the hdWGCNA package with a soft-thresholding power of 12. TF activity was inferred using decoupleR with the DoRothEA database. Cell–cell communication was analyzed using CellChat with the CellChatDB.mouse database.

### 4.5. Pseudotime Trajectory and Differentiation State Analysis

All acinar cells (*n* = 10,155) were extracted to construct a Monocle3 object. The cell closest to the centroid of all control cells was set as the trajectory root. The learn_graph function (use_partition = FALSE) was used to reconstruct the continuous transition trajectory from normal to injured states. Pseudotime values were calculated for each cell. LOESS local regression was applied to analyze the trend of cuproptosis scores along pseudotime, and the graph_test function was used for spatial autocorrelation testing. Additionally, CytoTRACE was used to assess the differentiation state (stemness) of acinar cells and its relationship with pseudotime.

### 4.6. External Validation Using Independent Datasets

The core findings from the single-cell analysis were cross-validated using the independent mouse AP model single-cell RNA-sequencing dataset GSE188819, reproducing the changes in cuproptosis pathway scores and expression patterns of core genes. For the human whole-blood transcriptomic dataset GSE194331, differential expression analysis was performed using the limma-voom method to compare cuproptosis-related gene expression between acute pancreatitis patients and healthy controls. Receiver operating characteristic (ROC) curves were plotted using the pROC package, and the area under the curve (AUC) was calculated as an exploratory analysis to assess the potential discriminatory ability of each cuproptosis-related gene. These analyses should be interpreted with caution because the dataset is derived from whole blood and may partly reflect circulating immune-cell composition.

### 4.7. Animal Experiments

Eight-week-old male C57BL/6 mice were obtained from the Nanjing Biomedical Research Institute of Nanjing University (Nanjing, China). Mice were randomly assigned to control (CON), caerulein-treated (SAP), and caerulein plus tetrathiomolybdate (TTM) treatment (SAP + TTM) groups. After one week of acclimatization, mice received ten consecutive intraperitoneal injections of caerulein (Sigma-Aldrich, St. Louis, MO, USA) at 300 μg/kg per injection at 1-h intervals to induce severe acute pancreatitis. For TTM intervention, a single intraperitoneal dose of TTM (10 mg/kg; Shanghai Aladdin Biochemical Technology Co., Ltd., Shanghai, China) was given 1 h before the first caerulein injection. All mice were euthanized 24 h after the final injection, and blood and pancreatic tissue samples were collected.

### 4.8. Histological Analysis

At the end of the experiment, mice were euthanized, and pancreata were excised. Pancreatic tissues were fixed in 4% paraformaldehyde (Aladdin, Shanghai, China), dehydrated through a graded ethanol series (Nanjing Chemical Reagent Co., Ltd., Nanjing, China), cleared in xylene (Nanjing Chemical Reagent Co., Ltd., Nanjing, China), embedded in paraffin (Leica Microsystems, Wetzlar, Germany), and sectioned using a microtome (Leica RM2235, Leica Microsystems, Wetzlar, Germany). Sections were deparaffinized, rehydrated, and stained with hematoxylin and eosin (H&E). Hematoxylin (Beyotime Biotechnology, Shanghai, China) and eosin (Beyotime Biotechnology, Shanghai, China) were used according to standard protocols. Slides were dehydrated and examined by light microscopy (Olympus IX73, Olympus Corporation, Tokyo, Japan).

### 4.9. Immunohistochemical Staining

At the end of the experiment, mice were euthanized and pancreata were excised. Pancreatic tissues were fixed in 4% paraformaldehyde (Aladdin, Shanghai, China), dehydrated, embedded in paraffin blocks (Leica Microsystems, Wetzlar, Germany), and sectioned. After deparaffinization and rehydration, sections underwent microwave antigen retrieval in citrate buffer (pH 6.0; Beyotime Biotechnology, Shanghai, China), cooled to room temperature, and were washed with phosphate-buffered saline (PBS; HyClone, Logan, UT, USA). Endogenous peroxidase was blocked with 3% hydrogen peroxide (Nanjing Chemical Reagent Co., Ltd., Nanjing, China), and non-specific binding was blocked with goat serum (Beyotime Biotechnology, Shanghai, China). Sections were incubated overnight at 4 °C with primary antibodies against FDX1 (Abcam, Cambridge, UK) and DLAT (Abcam, Cambridge, UK), then with HRP-conjugated secondary antibodies (Abcam, Cambridge, UK). DAB chromogen (Beyotime Biotechnology, Shanghai, China) was used for color development. Nuclei were counterstained with hematoxylin (Beyotime Biotechnology, Shanghai, China). Sections were examined under a light microscope (Olympus IX73, Olympus Corporation, Tokyo, Japan).

### 4.10. Immunofluorescence Staining

Immunofluorescence staining followed standard protocols. For cellular staining, cells were fixed with 4% paraformaldehyde (Aladdin, Shanghai, China) and permeabilized with 0.2% Triton X-100 (Sigma-Aldrich, St. Louis, MO, USA). After blocking with 5% bovine serum albumin (BSA; Beyotime Biotechnology, Shanghai, China), cells were incubated overnight at 4 °C with primary antibodies against DLAT (Abcam, Cambridge, UK) and FDX1 (Abcam, Cambridge, UK). After washing with PBS (HyClone, Logan, UT, USA), cells were incubated with Alexa Fluor 594- or 488-conjugated secondary antibodies (Invitrogen, Thermo Fisher Scientific, Waltham, MA, USA) and counterstained with DAPI (Wuhan Google Biotechnology Co., Ltd., Wuhan, China). Images were captured with an Olympus inverted fluorescence microscope (IX73, Olympus Corporation, Tokyo, Japan) and analyzed using ImageJ (version 1.53t).

### 4.11. Western Blot

Total protein was extracted from mouse pancreatic tissues. Protein concentrations were measured by BCA assay (Thermo Fisher Scientific, Waltham, MA, USA), and equal amounts were loaded per sample. Proteins were resolved by SDS-PAGE using reagents from Beyotime Biotechnology (Shanghai, China) and transferred to methanol-activated polyvinylidene difluoride (PVDF) membranes (MilliporeSigma, Burlington, MA, USA). Membranes were blocked with 5% non-fat milk (Beyotime Biotechnology, Shanghai, China) in TBST (Beyotime Biotechnology, Shanghai, China) for 1 h at room temperature, then incubated overnight at 4 °C with primary antibodies against FDX1 (Abcam, Cambridge, UK), DLAT (Abcam, Cambridge, UK), and GAPDH (Abcam, Cambridge, UK). After TBST washes, membranes were incubated with HRP-conjugated secondary antibodies (Abcam, Cambridge, UK) for 1 h at room temperature. Signals were developed with an ECL kit (Clinx Science Instruments Co., Ltd., Shanghai, China) and imaged using a chemiluminescence imaging system (Clinx Science Instruments Co., Ltd., Shanghai, China).

### 4.12. Cells and Cell Culture Conditions

The mouse pancreatic acinar cell line 266-6, high-glucose DMEM, fetal bovine serum (FBS), penicillin–streptomycin, phosphate-buffered saline (PBS), and 0.25% trypsin–EDTA were purchased from Shanghai Zhongqiao Xinzhou Biotechnology Co., Ltd. (Shanghai, China). Cells were thawed at 37 °C, resuspended in high-glucose DMEM supplemented with 10% FBS and 1% penicillin–streptomycin, and cultured in a humidified incubator at 37 °C with 5% CO_2_. At 80–90% confluence, medium was aspirated, cells were rinsed with PBS, and digested with 0.25% trypsin–EDTA at 37 °C for 1–2 min. Digestion was terminated with complete medium containing serum. Cells were subcultured at 1:2 to 1:4, with fresh medium every 2–3 days.

### 4.13. Inductively Coupled Plasma Mass Spectrometry (ICP-MS)

After the experiment, cell pellets or pancreatic tissue samples were washed three times with pre-cooled PBS (HyClone, Logan, UT, USA). Samples were transferred to digestion vessels, and ultra-pure concentrated nitric acid (Nanjing Chemical Reagent Co., Ltd., Nanjing, China) was added for overnight pre-digestion. Microwave digestion was performed using a CEM MARS 6 system (CEM Corporation, Matthews, NC, USA) with a programmed temperature ramp until clear. Digests were evaporated to near-dryness at 120–140 °C, cooled, and reconstituted with ultra-pure water from a Milli-Q system (MilliporeSigma, Burlington, MA, USA). Copper standard solution (Sigma-Aldrich, St. Louis, MO, USA) and internal standard (Sigma-Aldrich, St. Louis, MO, USA) were prepared. After optimizing the ICP-MS system (Agilent 7900, Agilent Technologies, Santa Clara, CA, USA), copper ion counts were measured, and copper concentration was calculated from the standard curve, expressed per milligram of protein or per gram of wet tissue.

### 4.14. Flow Cytometry

After treatment, cells were collected, digested with trypsin (Gibco, Thermo Fisher Scientific, Waltham, MA, USA), centrifuged, washed twice with PBS (HyClone, Logan, UT, USA), and resuspended in binding buffer or serum-free medium at 1 × 10^6^ cells/mL. A 100 μL aliquot was transferred to a flow cytometry tube, and probes were added: Annexin V/PI for apoptosis (Roche Diagnostics, Mannheim, Germany), DCFH-DA for ROS (Beyotime Biotechnology, Shanghai, China), JC-1 for mitochondrial membrane potential (Beyotime Biotechnology, Shanghai, China), and MitoSOX for mitochondrial ROS (Invitrogen, Thermo Fisher Scientific, Waltham, MA, USA). Samples were mixed and incubated at room temperature in the dark for 15–30 min. Reactions were terminated with buffer, filtered, and analyzed on a flow cytometer (BD FACSCanto II, BD Biosciences, Franklin Lakes, NJ, USA).

### 4.15. Transmission Electron Microscopy

After treatment, cells were collected, digested with trypsin (Gibco, Thermo Fisher Scientific, Waltham, MA, USA), and centrifuged at 1000 rpm for 5 min. The supernatant was discarded, and the pellet was fixed overnight at 4 °C with 2.5% glutaraldehyde (Aladdin, Shanghai, China) in 0.1 M phosphate buffer. Samples were post-fixed with 1% osmium tetroxide (Aladdin, Shanghai, China) at 4 °C in the dark for 1–2 h. After PBS rinses (HyClone, Logan, UT, USA), cells were dehydrated through graded ethanol (Nanjing Chemical Reagent Co., Ltd., Nanjing, China) and acetone (Nanjing Chemical Reagent Co., Ltd., Nanjing, China), then infiltrated with embedding medium-acetone mixtures and pure embedding medium (Leica Microsystems, Wetzlar, Germany) overnight. Samples were polymerized at 60–65 °C for 48 h. Ultrathin sections were cut with an ultramicrotome (Leica EM UC7, Leica Microsystems, Wetzlar, Germany), collected on copper grids, stained with uranyl acetate (Aladdin, Shanghai, China) and lead citrate (Aladdin, Shanghai, China), and imaged with a transmission electron microscope (Hitachi HT7800, Hitachi High-Tech Corporation, Tokyo, Japan) at 80–100 kV.

### 4.16. Statistical Analysis

All data analyses were performed using R software (version 4.1.1; R Foundation for Statistical Computing, Vienna, Austria; https://www.r-project.org/). Comparisons between groups used the Wilcoxon rank-sum test (non-normally distributed data) or Student’s *t*-test (normally distributed data). Correlation analyses used Spearman’s rank correlation coefficient. Multiple comparisons were adjusted using the Benjamini–Hochberg method, with adjusted *p* < 0.05 considered statistically significant. Additional analyses were performed using SPSS (version 26.0; IBM Corp., Armonk, NY, USA), and graphs were generated with GraphPad Prism (version 8.0; GraphPad Software, San Diego, CA, USA).

## 5. Conclusions

This study systematically delineates the landscape of a cuproptosis-related program in pancreatic acinar cells at single-cell resolution and identifies a cuproptosis-related phenotype in acute pancreatitis. A cuproptosis-related program was selectively upregulated in acinar cells during acute pancreatitis, as evidenced by copper ion accumulation and coordinated upregulation of ferredoxin 1 (FDX1) and dihydrolipoamide S-acetyltransferase (DLAT) proteins; this program progressively intensified with the progression of acinar cell injury. In vitro and in vivo experiments further demonstrated that the copper chelator tetrathiomolybdate (TTM) attenuated these alterations, alongside amelioration of mitochondrial dysfunction and oxidative stress injury. In a human whole-blood transcriptomic cohort, the expression levels of cuproptosis-related genes exhibited a graded association with disease severity; however, these findings are exploratory, may partly reflect circulating immune-cell populations, and require further validation before any clinical predictive utility can be established. Collectively, this study provides a new perspective on the molecular mechanisms of acinar cell injury in acute pancreatitis and provides a theoretical basis for further investigation of therapeutic strategies targeting cuproptosis-related copper dyshomeostasis.

## Data Availability

The original contributions presented in this study are included in the article and Supplementary Materials. Further inquiries can be directed to the corresponding authors.

## Supplementary Materials

The following supporting information can be downloaded at: https://www.mdpi.com/article/10.3390/ijms27209004/s1.

## Abbreviations

The following abbreviations are used in this manuscript:

AP	Acute pancreatitis
SAP	Severe Acute Pancreatitis
AP-1	Activator protein-1
FDX1	Ferredoxin 1
DLAT	Dihydrolipoamide S-acetyltransferase
TTM	Tetrathiomolybdate
GSH	Glutathione (reduced)
GSSG	Glutathione (oxidized)
ROS	Reactive oxygen species
ER	Endoplasmic reticulum
TCA	Tricarboxylic acid
TNF-α	Tumor necrosis factor-alpha
IL-1β	Interleukin-1 beta
IL-6	Interleukin-6
MPO	Myeloperoxidase
SOD	Superoxide dismutase
MDA	Malondialdehyde
4-HNE	4-Hydroxynonenal
NLRP3	NOD-like receptor protein 3
cGAS-STING	Cyclic GMP-AMP synthase-stimulator of interferon genes
TF	Transcription factor
scRNA-seq	Single-cell RNA sequencing
UMAP	Uniform manifold approximation and projection
WGCNA	Weighted gene co-expression network analysis
ICP-MS	Inductively coupled plasma mass spectrometry
GAPDH	Glyceraldehyde-3-phosphate dehydrogenase
PVDF	Polyvinylidene fluoride
ROC	Receiver operating characteristic
AUC	Area under the curve
