# Supplementary material for: Integrated Single-Cell and Transcriptomic Analysis Suggests a Cuproptosis-Related Phenotype and Potential Therapeutic Target in Acute Pancreatitis
Source: Int J Mol Sci. 2026 Oct 11;27(20):9004. doi: 10.3390/ijms27209004 (PMC13705439; doi:10.3390/ijms27209004)
Supplement: Supplementary file 1 [file ijms-27-09004-s001.zip › Supplementary Materials.pdf]

Supplementary Results

S1. Exploratory WGCNA identifies modules associated with a cuproptosis-related signature

To explore the co-expression network architecture associated with cuproptosis-related genes in acute pancreatitis, we applied high-dimensional weighted gene co-expression network analysis (hdWGCNA) to construct a gene co-expression network. Soft-thresholding analysis showed that a power of β = 12 yielded a high scale-free fit index ($R^2 > 0.85$) while preserving mean connectivity, thereby satisfying the scale-free topology criterion and justifying subsequent module identification (Figure S1A).

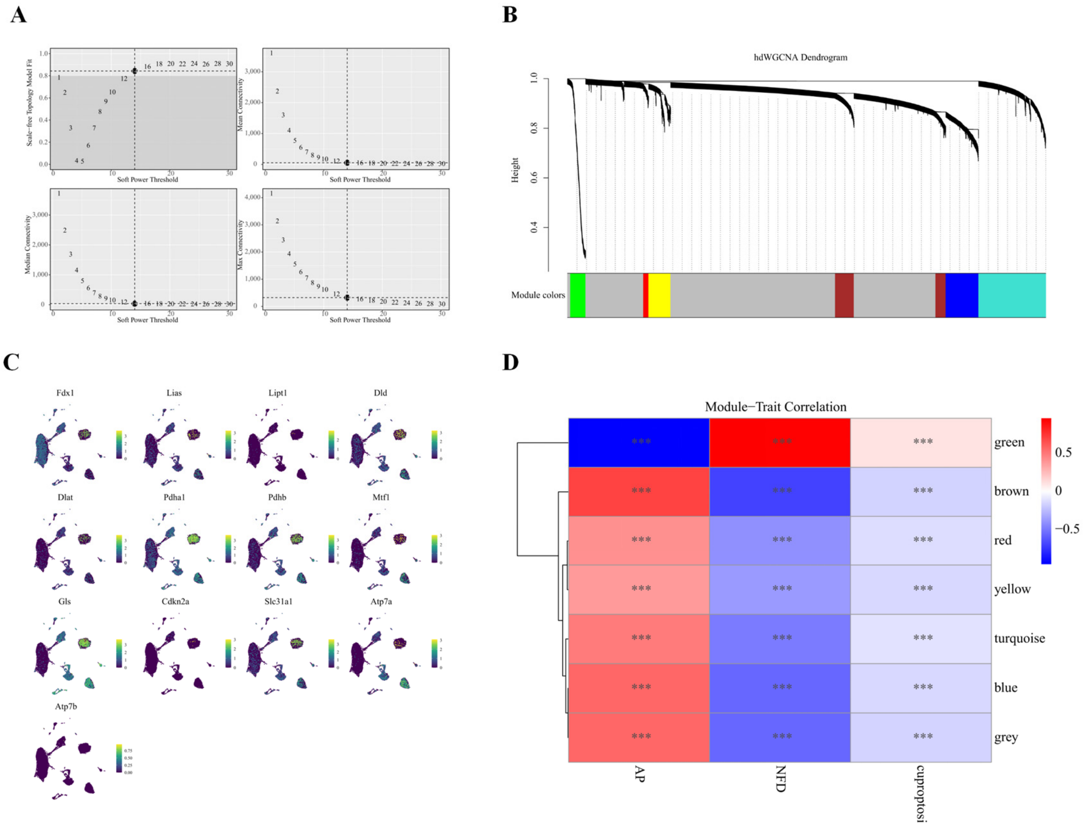

Figure S1. Exploratory co-expression network analysis of cuproptosis-related genes.**(A)** Soft-thresholding diagnostic plot for determining the optimal soft-thresholding power in weighted gene co-expression network analysis (WGCNA). The horizontal axis represents the soft-thresholding power, while the left vertical axis indicates the scale-free fit index and the right vertical axis represents the mean connectivity.**(B)** Gene clustering dendrogram based on hierarchical clustering of genes using the topological overlap matrix. The vertical axis denotes the distance between modules, and the different colour bands represent co-expression modules identified by the dynamic tree-cut algorithm. **(C)** UMAP dimensionality reduction plot overlaid with the eigengene activity scores of each module, with colours representing different modules, showing the enriched regions of each module in UMAP space. **(D)** Module–trait correlation heatmap displaying the Spearman correlation coefficients between each co-expression module and AP status, NFD status, and cuproptosis score. Red indicates positive correlation, blue indicates negative correlation, and the colour intensity represents the strength of correlation. Statistical significance levels are marked with asterisks.

With this soft threshold, we built a co-expression network from the 6,101 highly variable genes and performed hierarchical clustering based on the topological overlap matrix. The gene dendrogram displayed the expected hierarchical organization by expression similarity; the dynamic tree-cutting algorithm identified 10 co-expression modules, denoted by distinct colour bands (Figure S1B). UMAP visualization overlaid with module eigengene activity scores revealed that the modules occupied distinct regions of the UMAP space, and several modules exhibited pronounced activity enrichment in particular cell subsets, implying preferential association with specific cell types or functional states (Figure S1C). The module-trait correlation heatmap suggested that the red and brown modules were positively correlated with both AP status and the cuproptosis-related signature score (Spearman correlation coefficient > 0.6, $p < 0.001$), suggesting an association with a cuproptosis-related signature during acute pancreatitis (Figure S1D). Closer inspection of the gene composition showed that the turquoise module was enriched for immune/inflammatory response genes, and the cuproptosis-related regulators *Gls* (encoding glutaminase) and *Atp7a* (encoding a copper-transporting ATPase) both mapped to this module, suggesting potential transcriptional co-regulation between cuproptosis-related and inflammatory programmes. Additionally, the upstream cuproptosis-related regulator Fdx1 was found exclusively in the blue module, which was mainly enriched for ribosomal protein genes, suggesting that *Fdx1* may be associated with AP pathogenesis through an independent transcriptional program. These observations are exploratory and require further validation

S2. Exploratory transcriptional regulatory analysis of a cuproptosis-related program

To explore potential upstream transcriptional regulatory associations of a cuproptosis-related program, we collected known transcription factors (TFs) with predicted regulatory links to core cuproptosis-related genes from the DoRothEA database (evidence levels A, B, and C) and inferred single-cell TF activity using the decoupleR tool. These analyses are exploratory and hypothesis-generating and should not be interpreted as direct evidence of causality. A heatmap of the average activity of the top 30 most variable TFs in acinar cells, grouped by cell type, showed extensive differences in TF activity between the AP and NFD groups, with several TFs showing markedly increased or decreased activity in the AP group, suggesting possible global transcriptional reprogramming of acinar cells under AP conditions (Figure S2A).By integrating TFs with predicted regulatory links to the 13 core cuproptosis-related genes from DoRothEA, we identified 11 TFs with potential regulatory relationships with cuproptosis-related genes. A heatmap of the average activity distribution of these 11 TFs across cell types suggested cell-type-specific activity patterns: Spi1 showed the highest activity in macrophages and neutrophils, whereas Myc and Ppara were relatively more active in acinar and ductal cells, suggesting that cuproptosis-related transcriptional regulation may be cell-type-dependent (Figure S2B). A horizontal bar plot of the activity differences of these 11 cuproptosis-related TFs between AP and NFD acinar cells suggested that Spi1 (PU.1) activity was elevated in the AP group and reached statistical significance (adjusted $p < 0.05$); its predicted regulatory links to *Gls* and *Mtf1* were among the most well-defined, raising the possibility that Spi1 may serve as a potential upstream regulator of a cuproptosis-related program. This remains a hypothesis requiring experimental validation. Furthermore, the activities of Fos and Jun (AP-1 complex) were also increased, consistent with the known activation of NF-κB in AP, and the AP-1 complex was predicted to regulate Pdha1 expression. Vdr activity was also elevated in the AP group. In contrast, Myc activity showed a slight downward trend in the AP group, possibly reflecting the suppression of

proliferative programmes. The remaining TFs, such as Hnf4a and Foxa2, did not exhibit significant changes (Figure S2C). These findings are exploratory and require further validation.

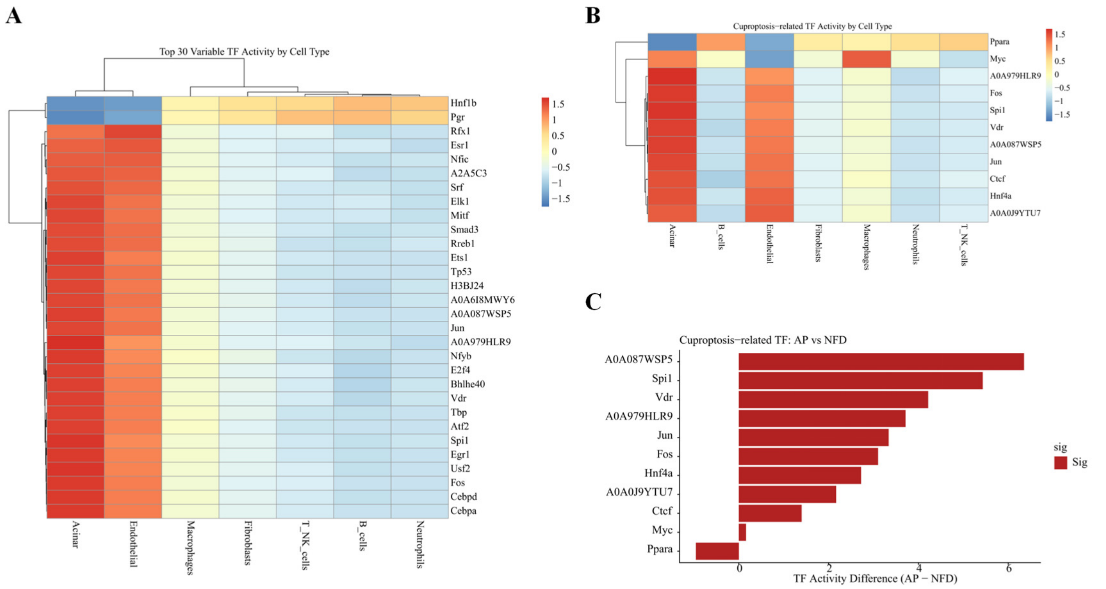

Figure S2. Exploratory transcriptional regulatory analysis of a cuproptosis-related program. **(A)** Heatmap showing the average activity of the top 30 transcription factors with the greatest variability in acinar cells, grouped by cell type. The color scale indicates differences in transcription factor activity among the groups. **(B)** Heatmap displaying the average activity distribution of transcription factors directly regulating core cuproptosis genes across various cell types. The horizontal axis represents different cell types, and the vertical axis represents transcription factors, with color intensity indicating the level of transcription factor activity. **(C)** Horizontal bar plot illustrating the differential activity of cuproptosis-related transcription factors in acinar cells between the AP and NFD groups, expressed as the difference (AP minus NFD). Red bars indicate positive differences, and blue bars indicate negative differences. Asterisks denote statistical significance levels.

S3. Exploratory analysis of cell－cell communication remodeling in acute pancreatitis

To explore how acinar cells with a cuproptosis-related signature may be associated with remodeling of the microenvironment through signaling molecules in acute pancreatitis, we employed the CellChat algorithm to compare intercellular signaling networks between the NFD and AP groups. These analyses are exploratory and hypothesis-generating and should not be interpreted as direct evidence of causality. The bar plot suggested that both the total communication strength and the number of communication events were greater in the AP group than in the NFD group (Figure S3A). A stacked bar plot of the relative activity of each signaling pathway in the NFD and AP groups showed that the proportional contributions of multiple inflammation- and injury-related pathways were altered in AP; among them, the MIF, CCL, Annexin, and Galectin pathways contributed substantially more, suggesting their potential involvement in remodeling intercellular communication under AP conditions (Figure S3B). The differential chord diagram comparing NFD and AP groups (left semicircle, NFD; right semicircle, AP) suggested that intercellular interactions were enhanced in the AP group, as reflected by more numerous and wider chords, suggesting a possible rise in overall communication activity in the acute pancreatitis state (Figure S3C). We next performed exploratory decomposition analysis on the signaling pathways whose activity was elevated in

the AP group. The MIF pathway chord diagram suggested that ligand–receptor interaction strength between macrophages and neutrophils was increased, raising the possibility that this pathway may contribute to the activation of innate immune cells (Figure S3D). The CCL chemokine pathway chord diagram displayed dense communication connections among multiple immune cell types, consistent with possible immune cell recruitment in acute pancreatitis (Figure S3E). The Annexin pathway chord diagram suggested that this pathway may primarily mediate communication between acinar cells and macrophages, and its alterations may be associated with the clearance of dead cells (Figure S3F). The Galectin pathway chord diagram similarly suggested predominant communication between acinar cells and immune cells, raising the possibility of a role in transmitting cell injury-related signals (Figure S3G). Collectively, these exploratory CellChat findings suggest that acinar cells with a cuproptosis-related signature may be associated with altered communication with neighboring immune and stromal cells through multiple signaling pathways; however, these analyses are inferential and hypothesis-generating and require experimental validation.

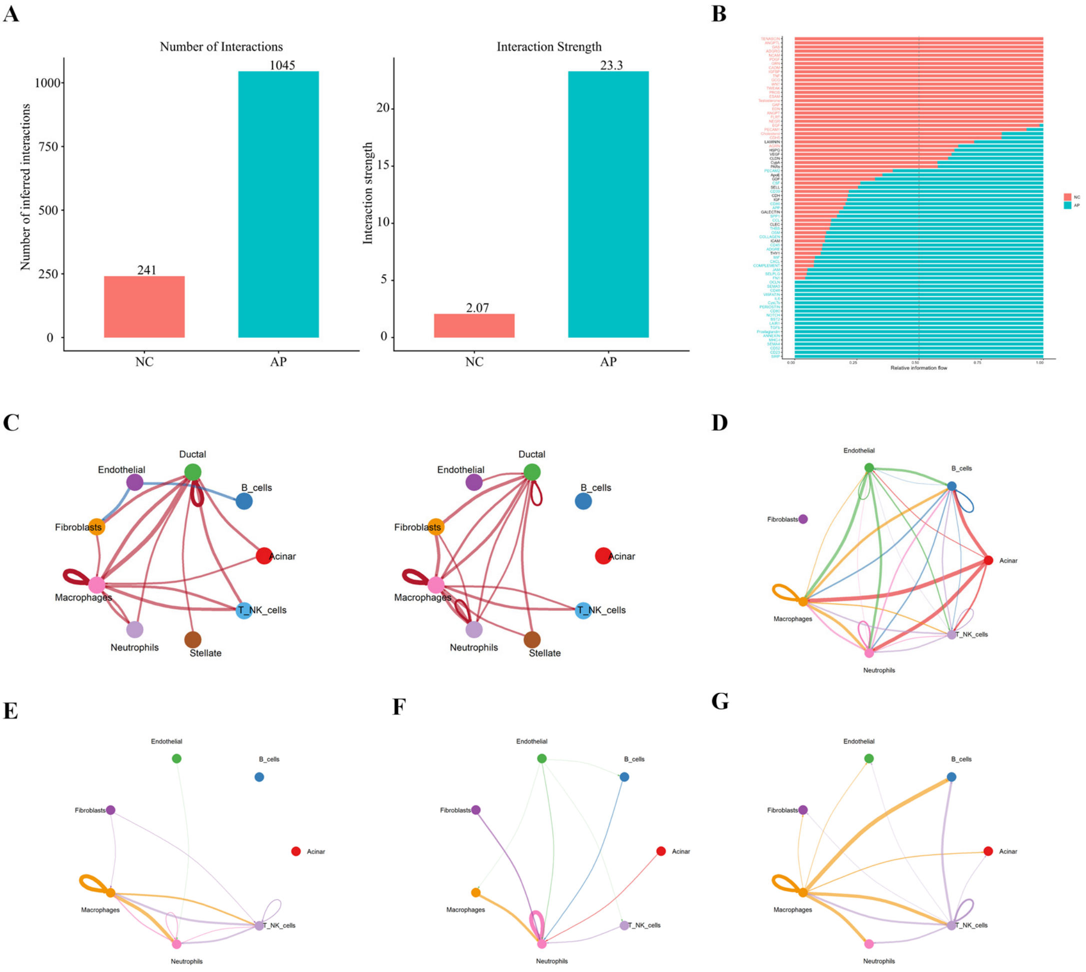

Figure S3. Exploratory cell–cell communication analysis in acute pancreatitis.**(A)** Bar plots comparing the number of intercellular communications (left) and total communication strength (right) between the NFD and AP groups. **(B)** Stacked bar plot showing the relative activity (contribution) of each signalling pathway in the NFD and AP groups, with the horizontal axis representing pathway names and the vertical axis representing the percentage of relative activity. **(C)** Chord diagram illustrating the differential communication network between the NFD and AP groups. The left semicircle represents the

NFD group, and the right semicircle represents the AP group; different colours denote different cell types, and the width of the chords indicates communication strength. **(D)** Chord diagram depicting the intercellular communication network of the MIF signalling pathway in the AP group, with different colours representing different cell types and chord width indicating the interaction strength of ligand–receptor pairs. **(E)** Chord diagram depicting the intercellular communication network of the CCL chemokine signalling pathway in the AP group. **(F)** Chord diagram depicting the intercellular communication network of the Annexin signalling pathway in the AP group. **(G)** Chord diagram depicting the intercellular communication network of the Galectin signalling pathway in the AP group.
